# Goldfish adiponectin: (I) molecular cloning, tissue distribution, recombinant protein expression, and novel function as a satiety factor in fish model

**DOI:** 10.3389/fendo.2023.1283298

**Published:** 2023-10-30

**Authors:** Yunhua Zheng, Cheng Ye, Mulan He, Wendy K. W. Ko, Ying Wai Chan, Anderson O. L. Wong

**Affiliations:** School of Biological Sciences, The University of Hong Kong, Hong Kong, Hong Kong SAR, China

**Keywords:** Adiponectin, food intake, feeding behavior, appetite control, goldfish

## Abstract

Adiponectin (AdipoQ) is an adipokine involved in glucose homeostasis and lipid metabolism. In mammals, its role in appetite control is highly controversial. To shed light on the comparative aspects of AdipoQ in lower vertebrates, goldfish was used as a model to study feeding regulation by AdipoQ in fish species. As a first step, goldfish AdipoQ was cloned and found to be ubiquitously expressed at the tissue level. Using sequence alignment, protein modeling, phylogenetic analysis and comparative synteny, goldfish AdipoQ was shown to be evolutionarily related to its fish counterparts and structurally comparable with AdipoQ in higher vertebrates. In our study, recombinant goldfish AdipoQ was expressed in *E. coli*, purified by IMAC, and confirmed to be bioactive via activation of AdipoQ receptors expressed in HepG2 cells. Feeding in goldfish revealed that plasma levels of AdipoQ and its transcript expression in the liver and brain areas involved in appetite control including the telencephalon, optic tectum, and hypothalamus could be elevated by food intake. In parallel studies, IP and ICV injection of recombinant goldfish AdipoQ in goldfish was effective in reducing foraging behaviors and food consumption. Meanwhile, transcript expression of orexigenic factors (NPY, AgRP, orexin, and apelin) was suppressed with parallel rises in anorexigenic factors (POMC, CART, CCK, and MCH) in the telencephalon, optic tectum and/or hypothalamus. In these brain areas, transcript signals for leptin receptor were upregulated with concurrent drops in the NPY receptor and ghrelin receptors. In the experiment with IP injection of AdipoQ, transcript expression of leptin was also elevated with a parallel drop in ghrelin mRNA in the liver. These findings suggest that AdipoQ can act as a novel satiety factor in goldfish. In this case, AdipoQ signals (both central and peripheral) can be induced by feeding and act within the brain to inhibit feeding behaviors and food intake via differential regulation of orexigenic/anorexigenic factors and their receptors. The feeding inhibition observed may also involve the hepatic action of AdipoQ by modulation of feeding regulators expressed in the liver.

## 1 Introduction

Adiponectin (AdipoQ), a member of the C1q/TNF-related protein family, is an adipokine produced by white adipose tissue and with high levels released into circulation (e.g., with 3–30 μg/ml in human serum and accounts for 0.01% of total plasma proteins) (1). The full-length AdipoQ is composed of an N-terminal signal peptide, a highly variable linker, a collagen-like domain, and a C-terminal globular domain. AdipoQ can exist in multiple forms, including the low molecular weight (LMW) trimers, moderate molecular weight (MMW) hexamers, and high molecular weight (HMW) oligomers (composed of 12-18 monomers) (2). These multimeric forms are produced by hydrophobic interactions among the globular domains, disulphide bonding via the Cys residue in the variable linker, and hydroxylation/glycosylation of Lys residues in the collagen-like domain (3). Through proteolytic cleavage in the collagen-like domain, a truncated form of trimeric AdipoQ composed of the globular domains, namely the globular AdipoQ (gAdipoQ), can also be generated (4). Different isoforms of AdipoQ have been identified in blood/at the tissue level (5) and confirmed to be bioactive in terms of receptor activation and biological functions (6). To date, two major types of AdipoQ receptors, AdipoR1 and AdipoR2, have been reported (7) and confirmed to be the members of the PAQR family, which is a unique family of membrane receptors with seven transmembrane domains similar to the G protein-coupled receptor but with opposite positioning of the N-terminal, C-terminal, and intra/extracellular loops (8). In mammals, the two receptors are expressed in a tissue-specific manner (e.g., with AdipoR1 expressed in muscle and AdipoR2 expressed in white fat and liver) (7), have distinct selectivity for different isoforms of AdipoQ (e.g., with AdipoR1 preferring LMW/gAdipoQ and AdipoR2 preferring HMW/MMW AdipoQ) (1), and mediate the biological actions of AdipoQ by functional coupling with the AMPK/PPAR, Ca^2+^/CaMKK, PI3K/Akt, MEK_1/2_/ERK_1/2_, JAK_2_/STAT_3_, and ceramide/sphingosine-dependent pathways (9–11). Although specific binding of T-cadherin with HMW/MMW but not LMW AdipoQ/gAdipoQ has been reported (e.g., in myoblasts) (12), the lack of transmembrane/intracellular structure for signal transduction in this cell adhesion molecule has raised the possibility that T-cadherin may act as a co-receptor to sequester HMW/MMW AdipoQ on the cell surface for subsequent AdipoR1/R2 activation (13).

Regarding the biological functions, AdipoQ is known to have pleiotropic actions in different tissues and involved in lipid and glucose metabolism (14, 15), energy homeostasis (16), adipocyte differentiation (17), insulin sensitization (18), cognitive function (19), bone mineralization/remodeling (20), pituitary hormone regulation (21), and tissue inflammation/immune responses (22). In humans, downregulation of serum AdipoQ can be associated with clinical cases of obesity (23), type 2 diabetes (24), cardiovascular disease (25), Alzheimer’s disease (26) and different types of cancer (e.g., breast cancer) (9). Furthermore, AdipoQ is well-documented to have antidiabetic, anti-obesogenic, anti-atherogenic, and anti-inflammatory effects (27), which forms the basis of the protective role of AdipoQ for a wide spectrum of diseases/pathological conditions, e.g., insulin resistance in type 2 diabetes (18), metabolic disorders associated with obesity (28), atherosclerosis and cardiovascular diseases (29), and kidney malfunction with albuminuria (30). As a result, AdipoQ has emerged as a biomarker as well as a new target for therapeutic intervention for various diseases in recent years (2).

Regarding the protective role of AdipoQ for obesity and diabetes, it has been largely attributed to the central effects of AdipoQ on feeding control, which can lead to reduction in body weight and body adiposity with improvement in glucose homeostasis (31). However, based on the studies in mammals (e.g., rodents), the effect of AdipoQ on food intake is highly controversial, as stimulatory (32), inhibitory (33), and no effect (34) have been reported [for a recent review on the controversy in feeding regulation by AdipoQ, see (35)]. For the stimulatory action reported (e.g., in mice), AdipoQ was shown to increase food intake via AdipoR1 coupled with AMPK activation within the hypothalamus (32). For the inhibitory action reported (e.g., in rats), AdipoR1 activation by AdipoQ in the hypothalamus could suppress food intake via the IRS_1_/Akt/FOXO_1_ and JAK_2_/STAT_3_ pathways (33). Apparently, the feeding regulation by AdipoQ is mediated by NPY and POMC neurons within the hypothalamus (36). In mice with feeding stimulation by AdipoQ, AdipoQ knockout could inhibit NPY with upregulation of POMC expression in the hypothalamus (32). In the same model with feeding inhibition by AdipoQ, AdipoQ treatment could induce hyperpolarization in NPY neurons with simultaneous excitation of POMC neurons in the hypothalamus (37). Interestingly, the excitation of POMC neurons by AdipoQ was found to be sensitive to glucose levels (38), suggesting that the energy status and/or macronutrient uptake may play a role in appetite control by AdipoQ. At present, other than NPY and POMC, not much is known regarding the role of other orexigenic/anorexigenic factors expressed within the hypothalamus/related-brain areas in feeding regulation by AdipoQ.

Although AdipoQ has been cloned in zebrafish (39), rainbow trout (40), ayu (41), yellow croaker (42), and more recently in sturgeon (43), except for the conflicting results for food deprivation on AdipoQ expression reported in zebrafish (39) and rainbow trout (40) and a marginal drop of food intake observed in sturgeon with AdipoQ treatment (43), little is known regarding the functional role or underlying mechanisms for feeding control by AdipoQ in fish models. In this study, goldfish was used as an animal model to examine the comparative aspects of AdipoQ as a feeding regulator in lower vertebrates. Goldfish was selected as it is widely used for feeding study in fish models (44) and by itself is also a well-documented representative of the carp family, the members of which account for ~51.1% of global finfish aquacultural production (data from 2022 FAO Report for World Fisheries & Aquaculture). As a first step, the structural identity and tissue expression profile of AdipoQ were established in goldfish by molecular cloning and RT-PCR. Based on the a.a. sequence obtained, the structural and evolutionary relationship between goldfish AdipoQ and its counterparts in other species was evaluated using sequence alignment, protein modeling, phylogenetic analysis, and comparative synteny. Since the research on fish AdipoQ has been hampered by a lack of homologous AdipoQ for functional studies, recombinant AdipoQ of goldfish origin was produced and confirmed to be bioactive via activation of goldfish AdipoR1/R2 expressed in HepG2 cells. Using the recombinant AdipoQ in feeding experiments with goldfish, we have provided evidence for the first time that AdipoQ could act as a novel satiety factor in fish models. In this case, the feeding regulation by AdipoQ was mediated by its central actions via differential modulation of feeding regulators and their receptors in brain areas involved in feeding control. Meanwhile, AdipoQ could also exert peripheral action to modify the feeding signals produced in the liver.

## 2 Materials and methods

### 2.1 Experimental animals

Goldfish (*Carassius auratus*) with a body weight of 29–38 g were imported from mainland Chain and acclimated in well-aerated water tanks under a 12-h light:12-h dark photoperiod at 20°C in our central aquarium facility for 4–5 weeks prior to any experimentation. Given that the fish used in our study were sexually regressed and sexual dimorphism was not apparent, goldfish of mixed sexes were used in our feeding experiments. In individual experiments, AdipoQ treatment and tissue sampling were performed according to the protocols (CULATR 5495-20) approved by the Committee for Animal Use in Teaching and Research at the University of Hong Kong.

### 2.2 Molecular cloning, sequence analysis, and tissue expression of goldfish AdipoQ

Goldfish AdipoQ (GenBank Accession No. ON087697) was cloned using 5′/3′ RACE as described previously (45) with primers designed according to the conserved regions of zebrafish AdipoQ using total RNA from goldfish brain as a template. Sequence alignment, protein modeling, and phylogenetic analysis were performed with Clustal W (http://www.ebi.ac.uk/clustalw), SWISS-MODEL (http://www.expasy.org/swissmod), and MEGA X (http://www.megasoftware.net/index.html), respectively. For analyses of intron/exon organization and associated comparative synteny, the AdipoQ genes together with their neighboring genes in the same genomic scaffold of representative species from different vertebrate classes were downloaded from NCBI genome database and curated with Splicing Finder and Genomicus software (https://www.genomicus.bio.ens.psl.eu/genomicus-57.01/). For tissue expression profiling of AdipoQ, RT-PCR was conducted in selected tissues and brain areas with primers and PCR conditions described in **Supplementary Table 1**. The authenticity of PCR products was confirmed using Southern blot with a DIG-labelled cDNA probe for goldfish AdipoQ and parallel RT-PCR for β actin was used as an internal control.

### 2.3 Production, characterization, and functional testing of recombinant goldfish AdipoQ

The coding sequence of the globular domain with a short fragment (6 a.a.) of the collagen-like domain of goldfish AdipoQ was PCR isolated and subcloned into the pTriEX6 expression vector (Merck KGaA, Darmstadt, Germany). The vector was transfected into Rosetta BL21 *E. coli* for expression of His-tagged gAdipoQ with IPTG induction as described previously (46). Goldfish gAdipoQ was used in our study as (i) gAdipoQ can penetrate the blood–brain barrier to induce biological functions via central action (31), and (ii) gAdipoQ, similar to the other isoforms, can effectively activate AdipoR1 and R2 expressed at the tissue level (1). The His-tagged gAdipoQ produced (referred to as goldfish AdipoQ) was purified by Immobilized Metal Affinity Chromatography (IMAC) using a Ni Sepharose 6 Fast Flow column (GE Healthcare, Chicago, IL) followed by size exclusion chromatography (SEC) using a HiLoad 26/60 Superdex 200 column (GE Healthcare). To determine the apparent MW of recombinant AdipoQ in solution, the top five fractions of the protein peak were pooled and a 200-µl sample was subjected to another round of SEC with multiangle laser light scattering (SEC-MALS) using a DynaPro NanoStar instrument (Wyatt Tech, Santa Barbara, CA) linked with a Superose 12 10/300 GL column (GE Healthcare) for sample loading. Meanwhile, the secondary structures (α helix/β sheet) and oligomeric status of goldfish AdipoQ were evaluated using far UV circular dichroism (CD) analysis with a Chirascan CD spectrometer (Applied Photophysics, Leatherhead, UK) and protein electrophoresis in native gel under non-denaturing conditions followed by Coomassie blue staining, respectively. To examine the bioactivity of goldfish AdipoQ produced, the ORFs of goldfish AdipoQ receptors, including AdipoR1a, AdipoR1b, and AdipoR2, were PCR-isolated and subcloned into the pcDNA/Zeo(−) expression vector (Thermo Fisher, Waltham, MA). These goldfish AdipoR expression vectors (80 ng each) were used in transfection studies in HepG2 cells with Lipofectamine (Thermo Fisher) for 6 h in serum-free Opti-MEM (Thermo Fisher) with 20 ng pEGFP, 20 ng pRL-TK, 300 ng PPRE_3_-TK-Luc reporter, and 20 ng PPAR_γ_ expression vector (with pcDNA/Zeo(−) as carrier DNA to make up a total of 500 ng DNA per transfection). The PPRE_3_-TK-Luc reporter carrying three copies of PPRE linked with the TK promoter is a firefly luciferase-expressing Luc reporter known to be responsive to AMPK activation under PPAR_γ_ expression (47). Following overnight recovery in serum-containing DMEM, HepG2 cells were treated with goldfish AdipoQ for 18 h to cause AMPK activation via individual isoforms of goldfish AdipoR (with treatment of metformin, an AMPK activator, as the positive control). After drug treatment, cell lysate was prepared in passive lysis buffer (Promega, Madison, WI) and subjected to luciferase activity measurement using a Dual-Glo™ Luciferase Assay Kit (Promega).

### 2.4 Raising antiserum and setting up fluorescence immunoassay for goldfish AdipoQ

Using the recombinant AdipoQ produced, antiserum for goldfish AdipoQ was raised in rabbit using the standard protocol in our lab (48). After initial immunization for the first month by subcutaneous injection of ~200 μg AdipoQ in Freund’s complete adjuvant (Sigma-Aldrich, St Louis, MO) followed by five rounds of booster injection once every 3 weeks with ~100 μg AdipoQ in Freund’s incomplete adjuvant (Sigma), final bleed was harvested and the specificity of antiserum obtained was tested by Western blot using protein lysates prepared from goldfish brain and liver, respectively (with parallel blotting of goldfish AdipoQ as a positive control). To further evaluate the ligand selectivity of the antiserum, a fluorescence immunoassay (FIA) was set up in protein A-coated 96-well FIA black plate (Corning, NY) after blocking with 10% BSA. In this assay, goldfish AdipoQ was biotinylated with an EZ-Link Sulfo-NHS-LC-Biotinylation Kit (Thermo Fisher) and used as the tracer. Samples to be tested (50 μl, with serial dilution of goldfish AdipoQ as the standards) were added into individual wells with 25 μl tracer and 25 μl AdipoQ antiserum (final dilution at 1:15K). After overnight incubation at 4°C, the content in individual wells was removed followed by adding 100 μl streptavidin-tagged horseradish peroxidase (SA-HRP, 0.5 μg/ml; Thermo Fisher). The FIA plate was then incubated at RT for 1 h with constant shaking followed by decanting of the SA-HRP solution. After washing with PBST buffer, the peroxidase activity in individual wells was measured using a QuantaBlu NS/K Fluorogenic Substrate Kit (Thermo Fisher). The ligand selectivity was validated by displacement studies using fish hormones with MW similar to AdipoQ, including growth hormone (GH), prolactin (PRL), somatolactin α/β (SLα/β), and leptin A/B. To evaluate the use of the assay in measuring AdipoQ released into systemic circulation, displacement study was also conducted with serial dilutions of plasma samples obtained from goldfish and grass carp, respectively.

### 2.5 *In vivo* studies for AdipoQ association with food intake and feeding regulation in goldfish

Goldfish were housed individually in 25-L water tanks and trained for ≥14 days prior to experimentation with a “one-meal-per-day” feeding schedule with ~2% BW of food pellets provided at 10:00 AM (as time zero for our experiments). For the study to test the effects of food intake on AdipoQ release in circulation and AdipoQ expression in selected tissues/brain areas, goldfish entrained with “one-meal-per-day” feeding schedule were divided into two groups, with one group receiving regular provision of food pellets (“Fed” group) and the other group without food provision (“Unfed” group). At different time points before/after the time for food provision, plasma samples, selected tissues (the liver, heart, muscle, and fat), and brain areas (the telencephalon, hypothalamus, and optic tectum) were harvested. For the study to examine the effects of AdipoQ on feeding behaviors, food intake, and gene expression of feeding regulators and their receptors, peripheral administration of AdipoQ were tested by intraperitoneal (IP) injection whereas the central effects of AdipoQ were evaluated by intracerebroventricular (ICV) injection of recombinant goldfish AdipoQ as described previously (49). In both cases, parallel injection with fish physiological saline was used as the control. IP (5 μl/g BW) and ICV injection (2 μl/fish) was conducted at 10 min and 15 min, respectively, prior to the time of food provision (at 10:00 AM). After that, the feeding behaviors in goldfish previously defined by Volkoff and Peter (50), including complete feeding (for surface foraging), bottom feeding (for bottom foraging), and incomplete feeding (for food spitting), were recorded using an AVD714 network surveillance system (AVTECH, Taiwan) and the three types of feeding behaviors were manually scored over a period of 2 h following AdipoQ treatment in a single-blind manner. By the end of the 2-h period, the food pellets remained were collected from individual tanks and dried to constant mass in a 65 °C oven. The mass difference of food pellets that remained versus the total amount added at the beginning of individual experiments was taken as the food consumption during the test period. For AdipoQ regulation of feeding regulators and their receptors expressed at the tissue level, the tissues and brain areas selected were harvested at different time points after IP/ICV injection of AdipoQ. Total RNA was isolated using TRIzol (Thermo Fisher), reversely transcribed by Superscript II (Invitrogen), and subjected to real-time PCR for target gene expression using a QuantiTect SYBR Green RT-PCR Kit (Qiagen, Hilden, Germany) with a Rotor-Gene Q PCR System (Qiagen). The PCR conditions and primers used for individual target genes are listed in **Supplementary Table 2**, and serial dilution of plasmid DNA with the ORF of target genes was used as the standards for calibration of target transcript expression. In our study, real-time PCR for 18S RNA was also conducted to serve as the internal control. To provide the anatomical basis for the central actions of AdipoQ, co-expression of AdipoQ and its receptors (including AdipoR1a, AdipoR1b, and AdipR2) in brain areas including the telencephalon, hypothalamus, and optic tectum was tested by RT-PCR using primers and PCR conditions as described in **Supplementary Table 1**. For central expression of the AdipoQ receptor at the protein level, Western blot was also conducted in goldfish brain lysate using the antisera for mouse AdipoR1 (1:1,000) and AdipoR2 (1:4,000), respectively (Aviva Systems Biology, San Diego, CA). The two antisera were raised in rabbit using peptide fragments (50 a.a. in size) specific for the respective receptors in mouse. Given that the two peptides also share 96% and 84% homology with the corresponding sequences in goldfish AdipoR1 and R2, respectively, the antisera are expected to have cross-reactivity with the target receptors in our fish model. In our study, the AdipoR1/R2 signals detected by Western blot were further confirmed by antibody preabsorption with peptide fragments used as the antigens for raising the respective antisera provided by the company and parallel blotting for β actin was used as an internal control.

### 2.6 Data transformation and statistical analysis

For functional validation of recombinant AdipoQ by activating AdipoR expressed in HepG2 cells, the firefly luciferase activity detected was normalized as a ratio of Renilla luciferase activity expressed in the same sample and transformed as a percentage of the mean value in the control group (as “%Ctrl”). For gene expression detected by real-time PCR in our *in vivo* studies, the raw data of transcript expression (in femtomole target transcript detected/μg RNA isolated) were normalized with the corresponding data of 18S RNA expression in the same sample and expressed as “%Ctrl” of the time-matched control. Data presented (Mean ± SEM; N = 6 for *in vitro* and 12 for *in vivo* experiments) were analyzed with one-way ANOVA followed by Newman–Keuls test for dose-dependence studies (with a single variable) or two-way ANOVA followed by Bonferroni test for time-course studies (with two variables) using Prism 6.0 (GraphPad, San Diego, CA). Differences between groups were considered significant at *p* < 0.05.

## 3 Results

### 3.1 Structural characterization and tissue distribution of goldfish AdipoQ

**Figure 1:**
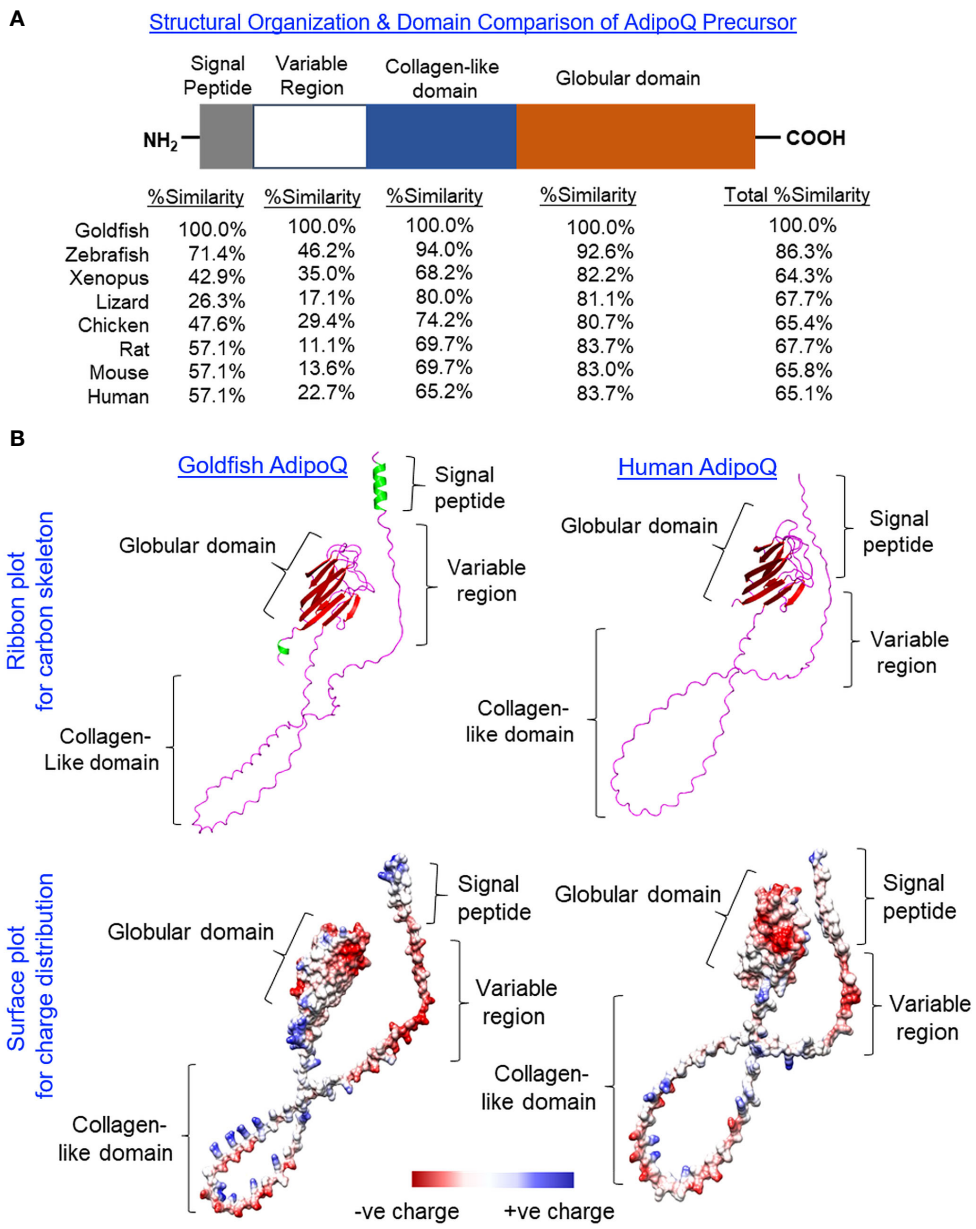
Domain organization and 3D protein modeling of goldfish AdipoQ. **(A)** Domain organization of the goldfish AdipoQ precursor compared with its counterparts in other vertebrates. The percentage similarity (%Similarity) for protein sequences of individual domains in the AdipoQ precursor of other species compared with the corresponding structures in goldfish AdipoQ were deduced by Clustal W with scoring > 0.5 using the Gonnet PAM250 matrix. **(B)** Comparison of 3D protein structure of goldfish AdipoQ precursor with its human counterpart. The ribbon plots for the 3D models of goldfish and human AdipoQ precursors were deduced by SWISS-MODEL with the anti-parallel β sheets labeled in red, α helical segments labeled in green, and random coil structures labeled in pink. The corresponding surface plots for charge distribution were constructed by Chimera 1.16 with the acidic residues carrying negative charge labeled in red, basic residues carrying positive charge labeled in blue, and hydrophobic residues with little/no charge labeled in white/light grey.

Using 3′/5′ RACE, the full-length cDNA of goldfish AdipoQ was cloned and sequence analysis reveals that it is composed of a 109-bp 5′ UTR, an 810-bp ORF, and a 314-bp 3′ UTR (**Supplementary Figure 1**). The ORF encodes a 269-a.a. precursor protein of goldfish AdipoQ (with deduced MW of 27.7 kDa) followed by the 3′ UTR with two putative polyadenylation signals (“tataaa” and “aatata”). Similar to its counterparts found in other vertebrates, the a.a. sequence of goldfish AdipoQ can be divided into the N-terminal signal peptide linked with a highly variable region followed by a collagen-like domain and the highly conserved C-terminal globular domain (**Figure 1A**). Based on sequence alignment (**Supplementary Figure 2**) coupled with domain comparison of AdipoQ precursors (**Figure 1A**), the protein sequences in the signal peptide (26.3%–71.4% homology) and variable region (11.1%–46.2% homology) of AdipoQ from different species appear to be quite diverse whereas the collagen-like domain (65.2%–94.0% homology) and C-terminal globular domain (80.7%–92.6% homology) are highly conserved from fish to mammals. As revealed by sequence alignment, the conserved residues/signature motifs of AdipoQ, e.g., the Cys residue close to the end of the variable region, the 22 G-X-Y repeats with three to four well-conserved Lys residues spreading within the collagen-like domain and the 10 anti-parallel β sheet structures in the globular domain, can still be noted in goldfish AdipoQ (**Supplementary Figure 2**). As shown by our 3D protein modeling (**Figure 1B**), the spatial clustering of the 10 β sheets into a jelly roll β barrel in the globular domain, the regular spacing of the 22 G-X-Y repeats in the collagen-like domain, and the overall charge distribution on the surface of AdipoQ molecule are also highly comparable between goldfish and human AdipoQ, although the helical structures found in the N-terminal signal peptide and C-terminal end of goldfish AdipoQ are absent in its human counterpart.

**Figure 2:**
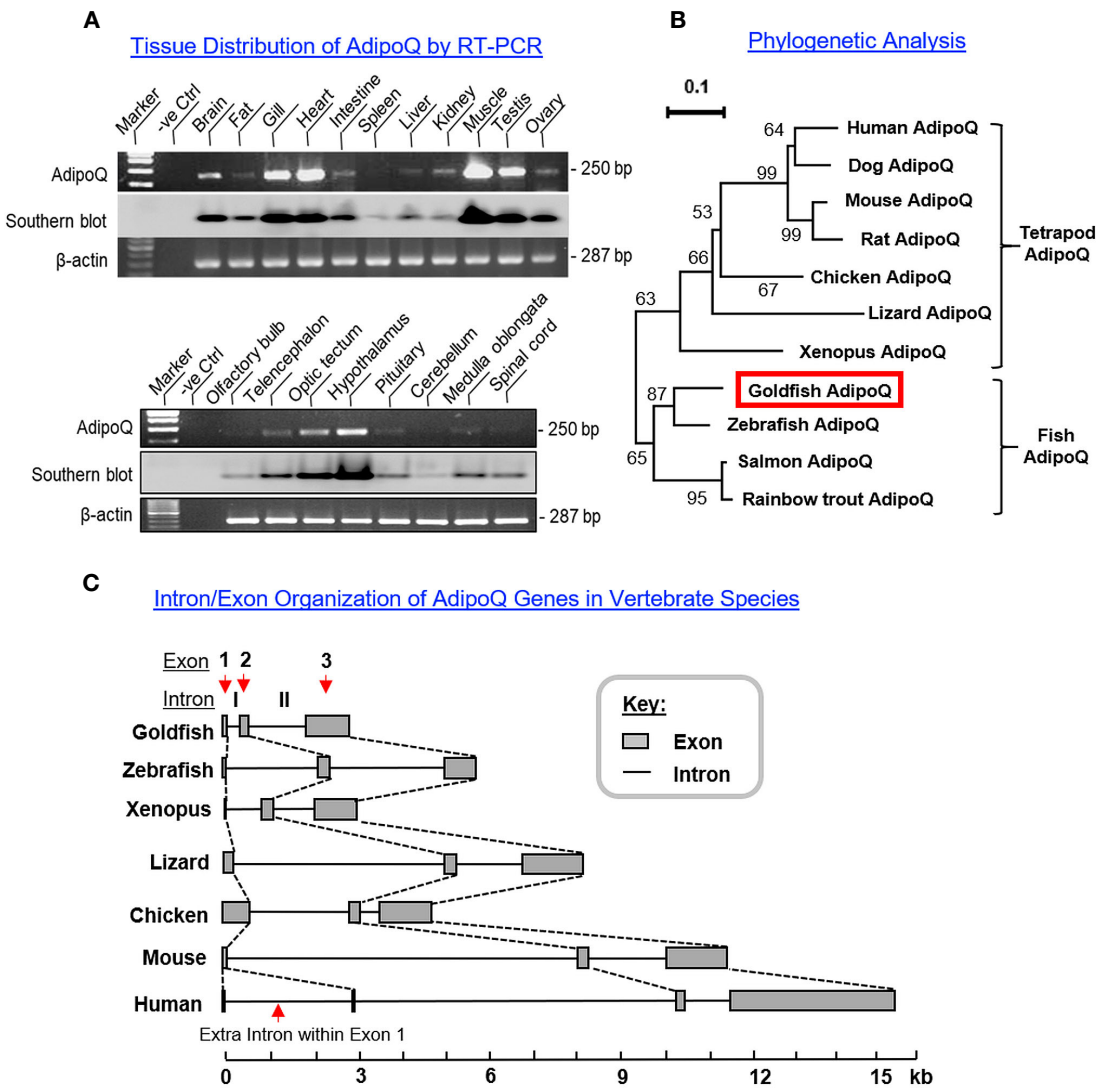
Tissue distribution, phylogenetic analysis, and intron/exon organization of goldfish AdipoQ. **(A)** Expression profiling of AdipoQ in different tissues and selected brain areas in goldfish using RT-PCR. The authenticity of PCR products detected was confirmed by PCR Southern using a DIG-labeled cDNA probe for goldfish AdipoQ and parallel PCR for β actin was used as an internal control. **(B)** Phylogenetic analysis of goldfish AdipoQ with the corresponding sequences found in other vertebrates using MEGA X with minimum evolution method. The guide tree was constructed with PHYLIP 2.0 with the percentage of bootstrap values (based on 1,000 bootstraps) presented in individual nodes, and the scale bar represents the phylogenetic distance of evolution. **(C)** Comparison of intron/exon organization in goldfish AdipoQ gene with the corresponding sequences in other vertebrates. The gene sequences for AdipoQ in representative species from fish to mammals were downloaded from the NCBI genome database and analyzed with Splicing Finder 2.4.1 to deduce the intron/exon junctions for structural comparison of AdipoQ genes in different species.

To shed light on the biological relevance of AdipoQ in different tissues, tissue expression profiling of AdipoQ was conducted in selected tissues and brain areas in goldfish using RT-PCR (**Figure 2A**). Unlike the findings in mammals with a tissue-specific expression pattern (5), AdipoQ was found to be ubiquitously expressed in the tissues/brain areas examined. At the tissue level, AdipoQ signals were highly expressed in the heart and muscle, to a lower extent in the gills and testis followed by the brain, and with a low to very low level of expression in other tissues including the liver, kidney, ovary, intestine, and fat (**Figure 2A**, upper panel). For the central expression of AdipoQ, the highest level of AdipoQ signal was found in the hypothalamus, to a lower extent in the optic tectum followed by telencephalon, and with rather low levels of expression in other brain areas including the olfactory bulb, pituitary, cerebellum, medulla oblongata, and spinal cord (**Figure 2A**, lower panel).

### 3.2 Phylogenetic analysis, intron/exon organization, and comparative synteny of AdipoQ genes

**Figure 3:**
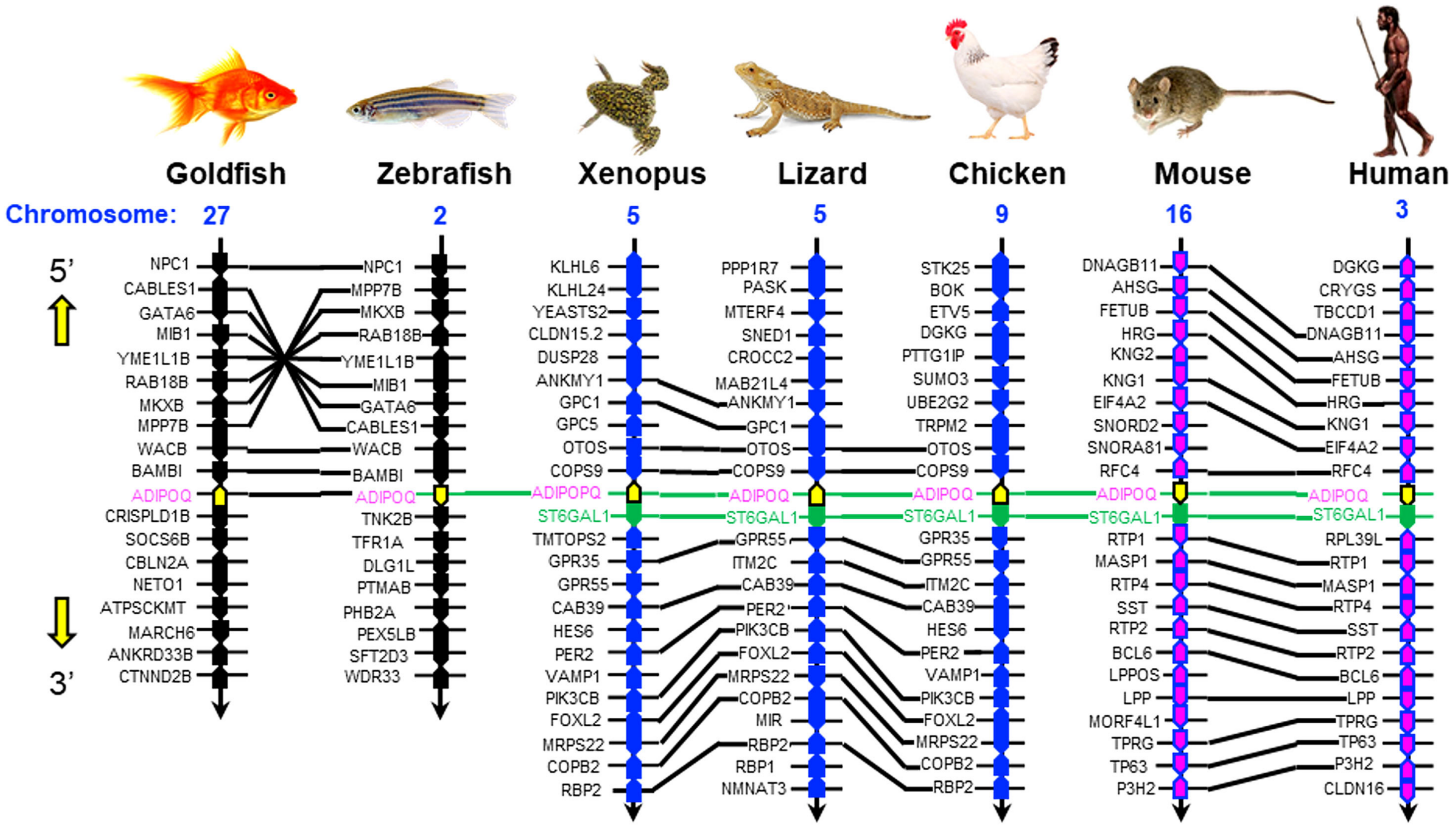
Comparative synteny of AdipoQ genes in the respective chromosomes from fish to mammals. The genomic scaffolds with AdipoQ gene in the respective chromosomes of different species were downloaded from the NCBI genome database. The neighboring genes located upstream/downstream of AdipoQ gene were curated using Genomicus software. Based on the similarity of syntenic genes around the AdipoQ gene in different species, three clusters of evolution lineages related to AdipoQ gene can be discerned, including the fish lineage (goldfish and zebrafish), amphibian/reptilian/avian lineage (Xenopus, lizard, and chicken), and mammalian lineage (mouse and human). The syntenic genes located in the neighborhood of AdipoQ gene in the same genomic scaffold are presented in the form of colored polygons with the pointed end indicating the transcriptional orientation. The ortholog genes in the same vertebrate class/evolution lineage are linked with black lines whereas the AdipoQ and ST6GAL1 genes, which can be found in different vertebrate classes/evolution lineages, are linked with green lines.

To establish the evolutionary relationship of goldfish AdipoQ with its counterparts in other vertebrates, phylogenetic analysis using the minimum evolution method was conducted using the gene sequence of goldfish AdipoQ together with the corresponding sequences in species from different vertebrate classes. As shown in **Figure 2B**, goldfish AdipoQ could be clustered in the clade of fish AdipoQ and with a close evolutionary relationship to the AdipoQ in zebrafish, which is also a member of the carp family. Based on the structural analysis of the full genes of AdipoQ in different species (**Figure 2C**), the intron/exon organization of AdipoQ genes (composed of two introns and three exons and with the ORF encoded in exons 2 and 3) was found to be well-conserved from fish to mammals with the exception that an extra intron was inserted into the original exon 1 in the human AdipoQ gene. Parallel analysis of the syntenic environment of AdipoQ genes in their respective genomic scaffold (**Figure 3**) also showed that the syntenic genes upstream/downstream of the AdipoQ gene were quite comparable in three separate vertebrate clusters, namely (i) the fish lineage (goldfish and zebrafish), (ii) the amphibian/reptilian/avian lineage (Xenopus, lizard, and chicken), and (iii) the mammalian lineage (mouse and human). Apparently, the AdipoQ gene in fish species had been translocated to a different gene locus in another chromosome during the evolution from fish to amphibian. When comparing the syntenic genes around AdipoQ in tetrapods, the ST6GAL1 gene was consistently found right after the AdipoQ gene despite the fact that the syntenic genes around AdipoQ between the mammals and the amphibian/reptilian/avian lineage are quite different. It is likely that a second translocation to a new genomic locus might have occurred in a gene fragment containing both the AdipoQ and ST6GAL1 genes during the evolution of the mammalian lineage.

### 3.3 Purification, characterization, and functional testing of recombinant goldfish AdipoQ

**Figure 4:**
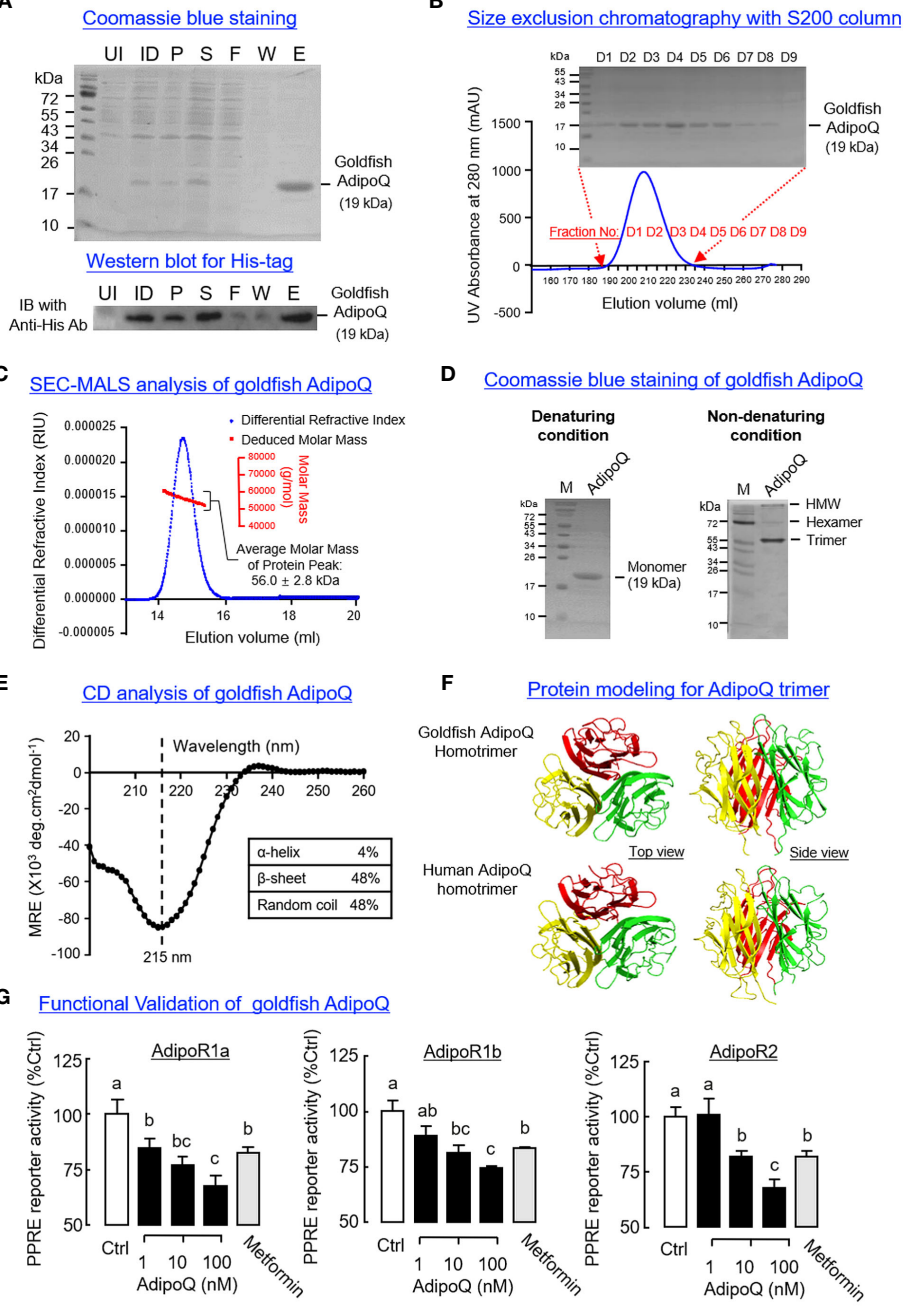
Recombinant goldfish AdipoQ expression, purification, characterization, and functional testing. **(A)** Expression of His-tagged goldfish AdipoQ in *E coli* followed by IMAC purification. Samples obtained at different stages of purification were resolved by SDS-PAGE followed by Coomassie blue staining. The identity of His-tagged AdipoQ expressed was confirmed by Western blot using an anti-His antibody (Anti-His Ab) (UI, uninduced bacteria culture; ID, bacteria culture induced with 1 mM IPTG; P, pellet fraction of induced culture; S, soluble fraction of induced culture; F, flow-through of soluble fraction after passing through IMAC column; W, Washout fraction with 60 mM imidazole; E, eluate with product released by 300 mM imidazole). **(B)** Size exclusion chromatography (SEC) for further purification of goldfish AdipoQ. Recombinant AdipoQ eluted from the IMAC column was loaded into a HiLoad 26/60 S200 SEC column, and the protein fractions covering the protein peak were then used for SDS-PAGE followed by Coomassie blue staining. **(C)** Evaluation of apparent MW in solution for recombinant AdipoQ using size exclusion chromatography coupled with multiangle laser light scattering (SEC-MALS). The top five protein fractions obtained from the preceding study were pooled, and a 200-μl sample of the final product was subjected to a second round of SEC using a S12 10/300 GL column linked with a DynaPro NanoStar Dynamic Light Scattering Detector. In this case, a protein peak with an average MW of 56 kDa (~3 folds of the AdipoQ monomer) could be detected by SEC-MALS analysis. **(D)** Characterization of recombinant AdipoQ with PAGE in native gel under non-denaturing conditions. Parallel SDS-PAGE under denaturing conditions was used as a reference for the monomeric form of AdipoQ. Under non-denaturing conditions, the trimeric, hexameric, and HMW forms of AdipoQ could be detected with the trimeric AdipoQ as the dominant product. **(E)** Structural analysis of recombinant goldfish AdipoQ using circular dichroism (CD). Goldfish AdipoQ produced was subjected to far UV CD analysis to evaluate the secondary structures (α helix/β sheet) found in goldfish AdipoQ (with subtraction of the background signal of the solvent used). **(F)** Protein modeling of the trimeric complex formed by the globular domains of goldfish AdipoQ compared with the corresponding structure in human. 3D protein modeling of goldfish gAdipoQ was constructed using SWISS-MODEL with the crystal structure of human gAdipoQ as a reference. **(G)** Function testing of recombinant AdipoQ in HepG2 cells with AdipoQ receptor (AdipoR) expression. After co-transfection of different components of the PPRE reporter system (for probing AMPK activation) with the expression vector for goldfish AdipoR (AdipoR1a, AdipoR1b, and AdipoR2, respectively), HepG2 cells were challenged for 18 h with increasing doses (1–100 nM) of recombinant AdipoQ and with parallel treatment of metformin (1 mM) as the positive control. After that, luciferase activities expressed were measured using a dual luciferase assay kit. Groups denoted with different letters represent a significant difference at *p* < 0.05 (one-way ANOVA followed by Newman–Keuls test).

To prepare the homologous AdipoQ in goldfish for functional testing, His-tagged protein of goldfish AdipoQ was expressed in *E. coli* and purified by IMAC (**Figure 4A**) followed by SEC (**Figure 4B**). The protein expressed (with MW of 19 kDa) was composed of the globular domain of goldfish AdipoQ with a short fragment of the collagen-like domain, and a similar preparation of recombinant AdipoQ in other species is known to activate AdipoR1/R2 (51) and trigger biological functions reported for AdipoQ (31). Using SEC-MALS, the recombinant protein prepared was found to have an apparent MW in solution of 56.0 ± 2.8 kDa (**Figure 4C**), which is around three times the MW detected for goldfish AdipoQ under SDS-PAGE with denaturing conditions (**Figures 4A, B**), implying that the recombinant AdipoQ prepared may exist in the form of a trimer. This idea is also supported by our finding that multiple forms of AdipoQ (including the HMW, hexameric and trimeric forms) could be detected in our AdipoQ preparation with native PAGE under non-denaturing conditions (**Figure 4D**). Of note, the trimeric form (with MW of 57.2 kDa, ~3 times of the monomer detected under denaturing conditions) was found to be the major product in our recombinant protein preparation.

According to the literature, the β-sheet structures of the globular domain of AdipoQ play a key role in trimer formation (e.g., in human gAdipoQ) (52). Based on our CD analysis, the goldfish AdipoQ prepared was confirmed to have a negative ellipticity minima at 215 nm and dominated by β-sheet structures (up to 48%) (**Figure 4E**). Similar to the human counterpart, the 3D modeling of the globular domain of goldfish AdipoQ revealed that the 10 anti-parallel β-sheets within individual globular domains could roll up into β barrels and form the hydrophobic surface to allow for the clustering of three gAdipoQ into a trimeric complex (**Figure 4F**). These results, as a whole, provide the structural basis for trimer formation in our preparation of recombinant AdipoQ. To confirm that the AdipoQ prepared is biologically active, functional expression of goldfish AdipoR1a, AdipoR1b, and AdipoR2 was performed in HepG2 cells with co-transfection of the PPARγ expression vector and PPRE_3_-TK-Luc reporter. The Luc reporter is responsive to AMPK induction with subsequent inhibition of the transcriptional activity mediated by PPRE via PPARγ activation (47). As shown in **Figure 4G**, increasing levels of recombinant goldfish AdipoQ (1–100 nM, 18 h) were effective in triggering a dose-dependent drop of PPRE-mediated Luc reporter activity in HepG2 cells with AdipoR1a, AdipoR1b and AdipoR2 expression, respectively. In these studies, a similar inhibition on Luc reporter activity was also observed with parallel treatment of the AMPK activator metformin (1 mM).

### 3.4 Characterization of AdipoQ antiserum and validation of goldfish AdipoQ FIA

Using the recombinant protein prepared, antiserum for goldfish AdipoQ was raised in rabbit. Titering of the antiserum by Western blot with goldfish liver lysate revealed that the antiserum with dilutions from 1:10K to 1:40K could detect a single band of AdipoQ immunoreactivity with MW of 53 kDa (**Supplementary Figure 3A**). By fixing the dilution at 1:15K, the antiserum not only could recognize the recombinant goldfish AdipoQ (19 kDa) but also detect the same 53-kDa protein band in tissue lysates from the liver and pituitary, respectively (**Supplementary Figure 3B**). In the same tissues, overnight preabsorption of the antiserum (1:40K) with goldfish AdipoQ (1 µM) was found to suppress the Western blot signals of the 53-kDa protein band (data not shown). Using a 1:15K dilution of AdipoQ antiserum, an FIA system with biotinylated goldfish AdipoQ as the tracer was set up to test for the ligand specificity of the antiserum. As shown in **Supplementary Figure 3C–E**, the specific binding of AdipoQ tracer could be displaced in a dose-dependent manner with increasing levels of recombinant goldfish AdipoQ (with ED_50_ of 37.1 ± 2.9 ng/ml and inter- and intra-assay CV of 11.4% and 7.2%, respectively). However, similar treatment with increasing doses of fish hormones with MW similar to AdipoQ, including common carp GH, goldfish PRL, grass carp leptin A and B, and grass carp SLα and SLβ, was not effective in this regard. To test for the use of the FIA system in quantitative measurement of AdipoQ release in systemic circulation, displacement study was also conducted with serial dilutions of plasma samples obtained from goldfish and grass carp, respectively (**Supplementary Figure 3F**). In both cases, a similar dose dependence for tracer displacement was noted with decreasing dilutions of the plasma samples with a clear parallelism compared with the displacement curve caused by increasing levels of goldfish AdipoQ. These results indicate that the FIA system can be used for measuring AdipoQ secretion in plasma samples of goldfish and grass carp.

### 3.5 Functional association of AdipoQ with food intake and feeding regulation in goldfish

**Figure 5:**
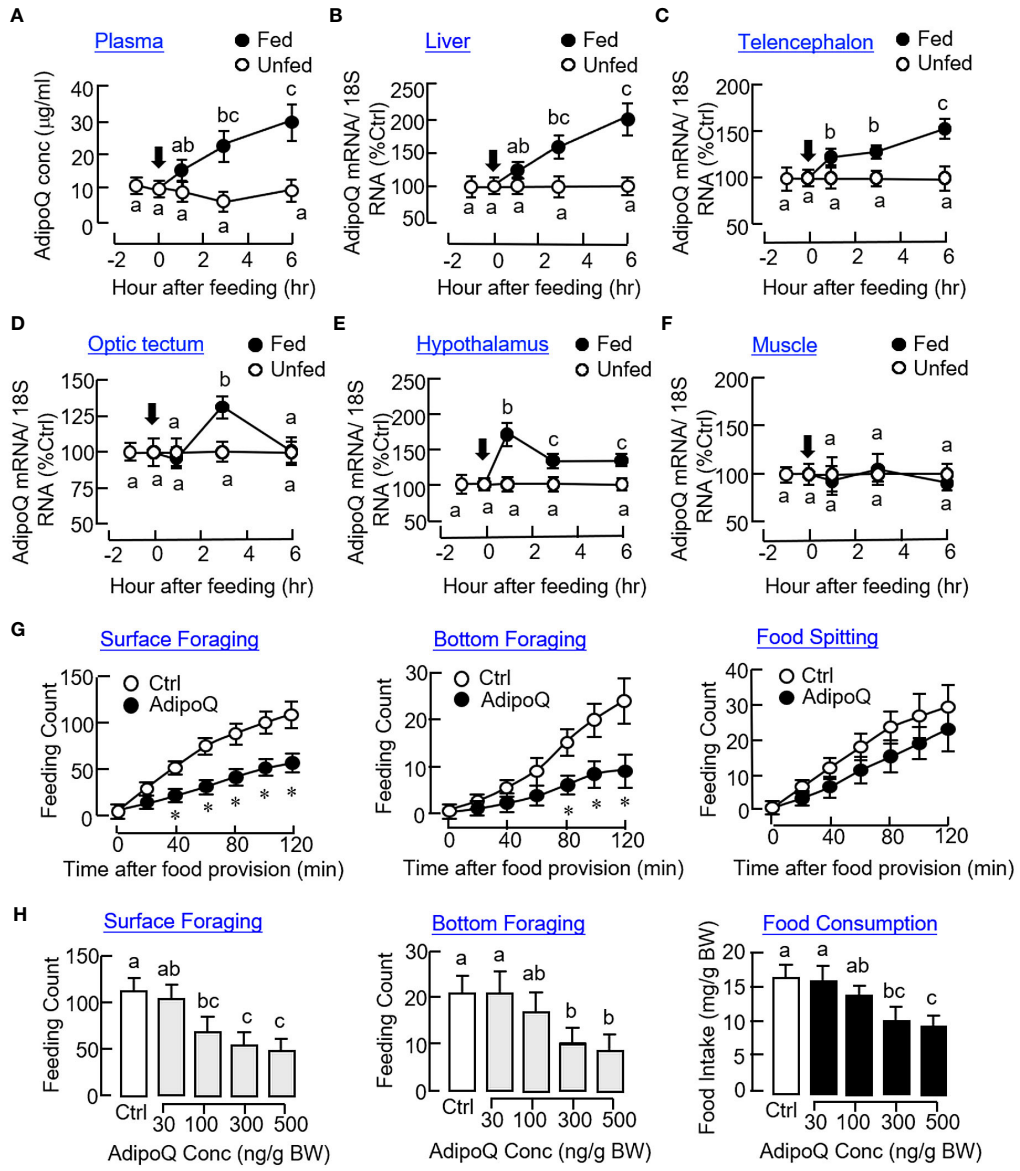
Association of AdipoQ with food intake and feeding regulation in goldfish. Effects of food intake in goldfish on plasma AdipoQ **(A)** and AdipoQ gene expression in the liver **(B)**, telencephalon **(C)**, optic tectum **(D)**, hypothalamus **(E)**, and muscle **(F)**, respectively. Goldfish entrained with a “one-meal-per-day” feeding schedule was divided into two groups, with one group as “Fed” group with food pellets provided at 10:00 AM (as time zero) and the other group as “Unfed” group without food provision (as the control). Plasma samples and selected tissues were harvested at the time points as indicated. Plasma AdipoQ was measured by FIA, and AdipoQ transcript level was monitored by real-time PCR. Time course **(G)** and dose dependence **(H)** of AdipoQ treatment on surface foraging, bottom foraging, and food spitting in goldfish. IP injection of recombinant goldfish AdipoQ (300 ng/g BW for time course and 100–500 ng/g BW for dose dependence) was conducted 10 min prior to the scheduled feeding time (at 10:00 AM), and different types of feeding behaviors were scored over a 2-h period after the introduction of food pellets. Parallel injection of physiological saline was used as the control. In the study for dose dependence of AdipoQ action, food consumption was also monitored by the end of the 2-h period. Groups denoted by asterisks (Student’s *t* test compared with time-matched control) or with different letters (two-way ANOVA followed by Bonferroni test for time course and one-way ANOVA followed by Newman–Keuls test for dose dependence study) represent a significant difference at *p* < 0.05.

As a first step to investigate the functional role of AdipoQ in goldfish feeding, the effect of food intake on AdipoQ expression were tested in goldfish entrained with a “one-meal-per-day” feeding schedule with food provision at 10:00 AM (“Fed” group) and with a parallel group without food provision to serve as the control (“Unfed” group). After initiation of food intake, plasma levels of AdipoQ measured by our newly established FIA system were elevated gradually from 1 h to 6 h (**Figure 5A**) with parallel rises in AdipoQ transcript levels in the liver (**Figure 5B**) and telencephalon (**Figure 5C**) up to 6 h. Meanwhile, a transient increase in AdipoQ mRNA was noted at 3 h in the optic tectum (**Figure 5D**). A similar rise in AdipoQ gene expression was also observed in the hypothalamus with peak at 1 h and reduced to a lower level above the basal up to 6 hr (**Figure 5E**). In the same study, food intake did not alter AdipoQ expression in muscle (**Figure 5F**) and the same was true for the corresponding responses in the pituitary, heart, and fat (data not shown). To shed light on the functional role of AdipoQ in goldfish feeding, IP injection with recombinant goldfish AdipoQ (300 ng/g BW) was also performed. As shown in **Figure 5G**, the major types of feeding behaviors observed in goldfish, namely the surface foraging and bottom foraging but not food spitting activity, were reduced in a time-dependent manner up to 2 h with AdipoQ treatment. By fixing the duration of treatment at 2 h, IP injection with increasing levels of recombinant AdipoQ (30–500 ng/g BW) was also effective in reducing the two types of foraging activities with a parallel drop in food consumption in a dose-related fashion (**Figure 5H**).

### 3.6 IP injection of AdipoQ on central expression of feeding regulators and their receptors

**Figure 6:**
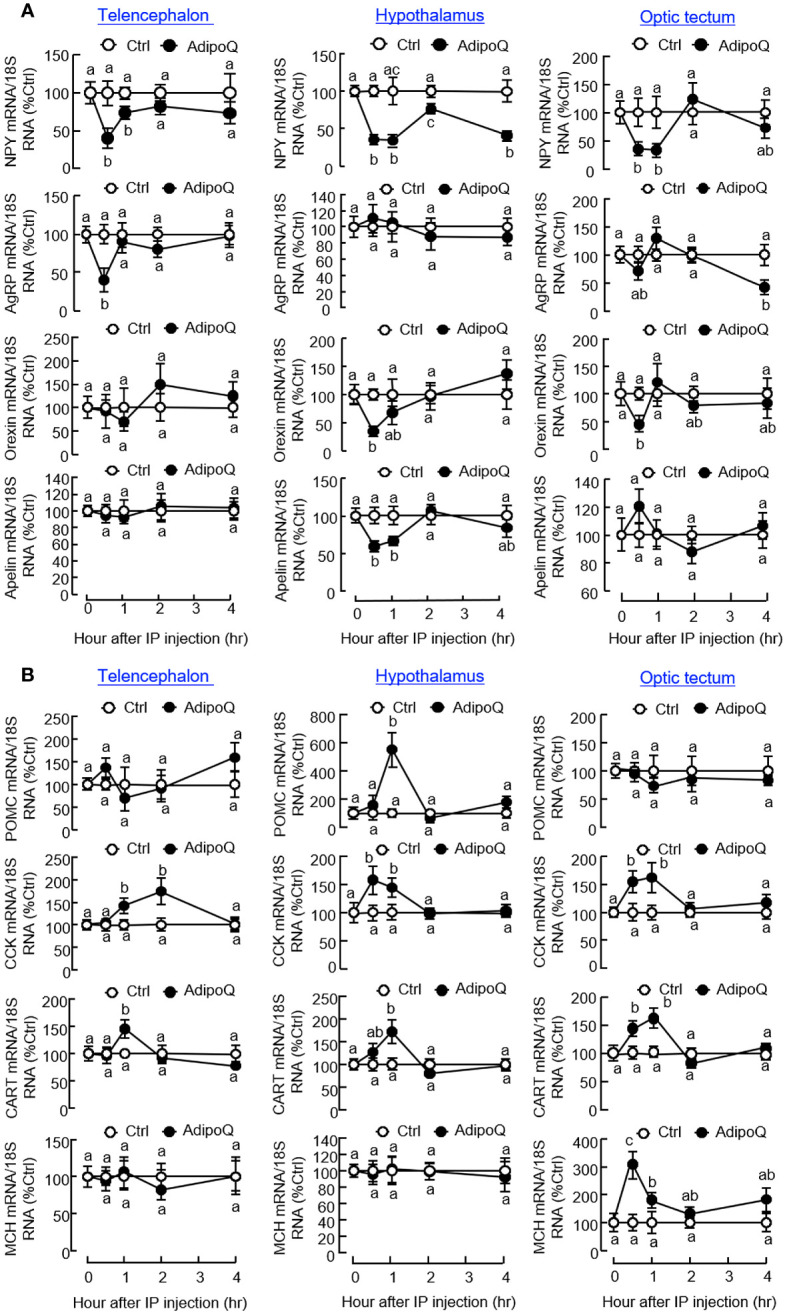
IP injection of AdipoQ on central expression of orexigenic/anorexigenic factors in brain areas for feeding control in goldfish. Goldfish with prior training of “one-meal-per-day” feeding schedule were IP injected with recombinant AdipoQ (300 ng/g BW) at the scheduled feeding time without food provision (as time zero). Parallel injection with physiological saline was used as the control. After IP injection, brain areas including the telencephalon, hypothalamus, and optic tectum were harvested at the time points as indicated. Total RNA was isolated and used for real-time PCR to monitor the transcript expression for **(A)** orexigenic factors including NPY, AgRP, Orexin, and Apelin, and **(B)** anorexigenic factors including CCK, POMC, CART, and MCH. Parallel real-time PCR for 18S RNA was used as the internal control. Groups denoted by different letters represent a significant difference at *p <* 0.05.

To unveil the mechanisms underlying the feeding inhibition by AdipoQ, IP injection of AdipoQ (300 ng/g BW) was conducted to examine its effects on feeding regulators and their receptors expressed in brain areas involved in feeding control. Regarding the orexigenic factors (**Figure 6A**), AdipoQ treatment was shown to reduce NPY transcript levels with peak inhibition from 0.5 h to 1 h in the telencephalon, hypothalamus, and optic tectum, and the drop in NPY signal at the hypothalamic level was maintained up to 4 h. During the first 0.5 h, transient drops in the transcript levels for AgRP in the telencephalon, orexin and apelin in the hypothalamus, and orexin in the optic tectum were also noted. Unlike the rapid responses observed in the telencephalon, a delayed inhibition on AgRP gene expression could be detected in the optic tectum at 4 h after AdipoQ treatment. For the anorexigenic factors expressed within these brain areas (**Figure 6B**), IP injection of AdipoQ was found to induce transient rises of CCK and CART transcripts in the telencephalon, hypothalamus, and optic tectum with peak from 0.5 h to 1 h (for hypothalamus and optic tectum) or even up to 2 h (for telencephalon). Similarly, other anorexigenic factors examined also showed transient rises in gene expression after AdipoQ treatment, with peak stimulation of MCH signal in the optic tectum at 0.5 h and POMC signal in the hypothalamus at 1 h, respectively.

**Figure 7:**
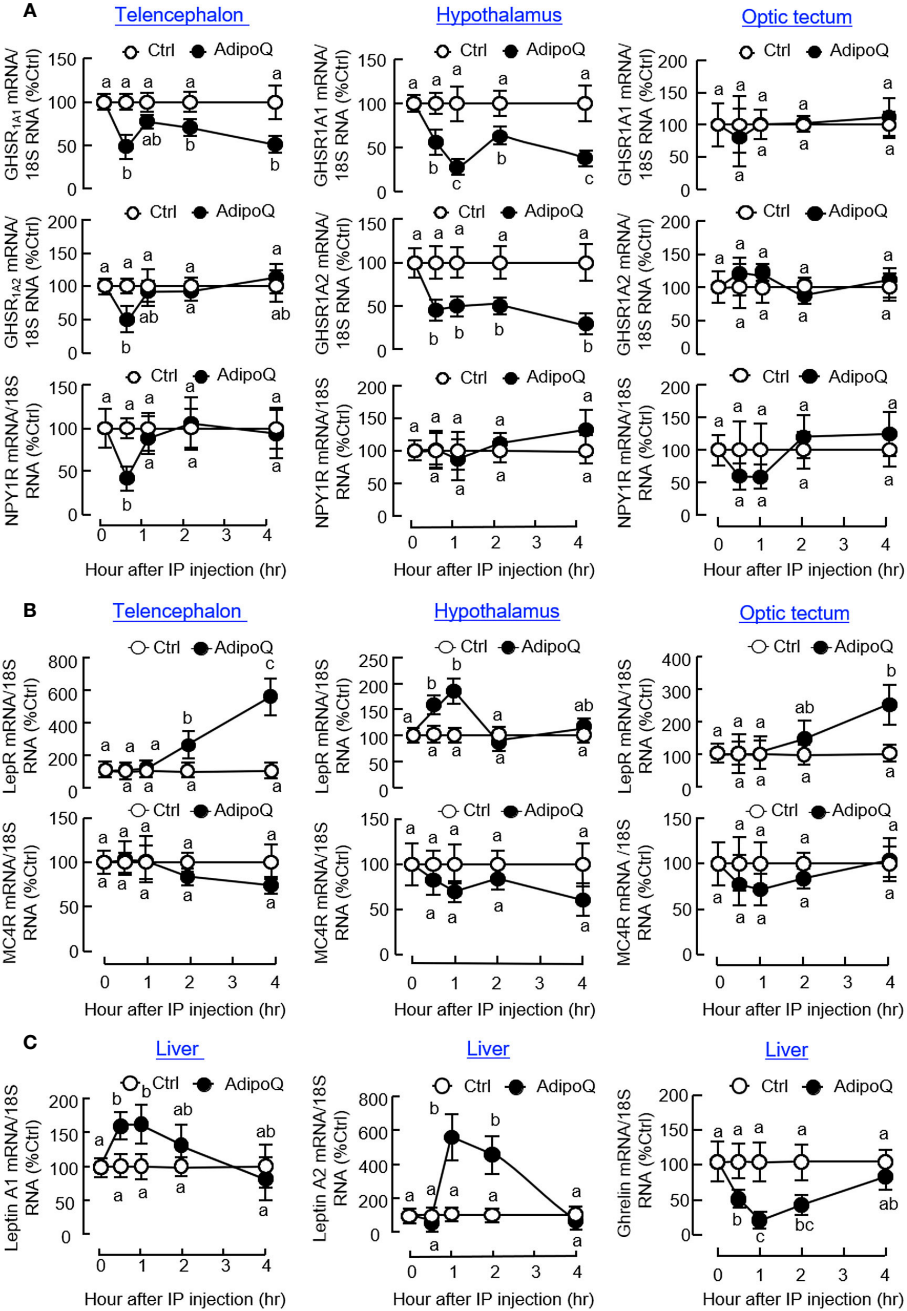
IP injection of AdipoQ on central expression of the receptors for feeding regulators and hepatic expression of leptin and ghrelin in goldfish. IP injection of AdipoQ (300 ng/g BW) was performed with parallel treatment of physiological saline as the control. After that, the liver as well as brain areas including the telencephalon, hypothalamus, and optic tectum were harvested at the time points as indicated. Total RNA was isolated from brain areas collected and used for real-time PCR to monitor transcript expression of **(A)** the receptors for orexigenic factors including GHSR_1A1_, GHSR_1A2_, and NPY1R, and **(B)** the receptors for anorexigenic factors including LepR and MC4R. Similar operation was conducted in the liver samples with real-time PCR for **(C)** the peripheral feeding regulators including leptin A1, leptin A2, and ghrelin. In this study, real-time PCR for 18S RNA was used as the internal control. Groups denoted by different letters represent a significant difference at *p <* 0.05.

Regarding the receptors for various feeding regulators, IP injection of AdipoQ could suppress transcript expression of the NPY receptor NPY1R in the telencephalon and ghrelin receptors, including GHSR_1A1_ and GHSR_1A2_, in the telencephalon and hypothalamus (**Figure 7A**). Unlike the rapid drops in NPY1R and GHSR_1A2_ signals detected at 0.5 h in the telencephalon, the corresponding inhibition on GHSR_1A1_ in the telencephalon and GHSR_1A1_ and GHSR_1A2_ in the hypothalamus was maintained up to 4 h. In contrast to the inhibitory actions on NPY/ghrelin receptors, AdipoQ treatment could also upregulate transcript expression of leptin receptor (LepR) in the hypothalamus (with a transient rise from 0.5 h to 1 h) as well as in the telencephalon and optic tectum (with a delayed elevation up to 4 h) but with no effect on the melanocortin receptor MC4R expressed in these brain areas (**Figure 7B**). In addition to the central effects on LepR and GHSR, differential regulation of their ligands at the hepatic level was also observed with IP injection of AdipoQ. In this case, AdipoQ treatment was effective in triggering transient rises in transcript expression of leptin A1 and leptin A2 in the liver (with peak responses at 1 h) but with parallel inhibition on ghrelin gene expression during the same period (**Figure 7C**). However, AdipoQ treatment did not alter transcript expression of insulin in the same tissue (data not shown).

### 3.7 Central expression of AdipoQ receptors and ICV injection of AdipoQ on goldfish feeding

**Figure 8:**
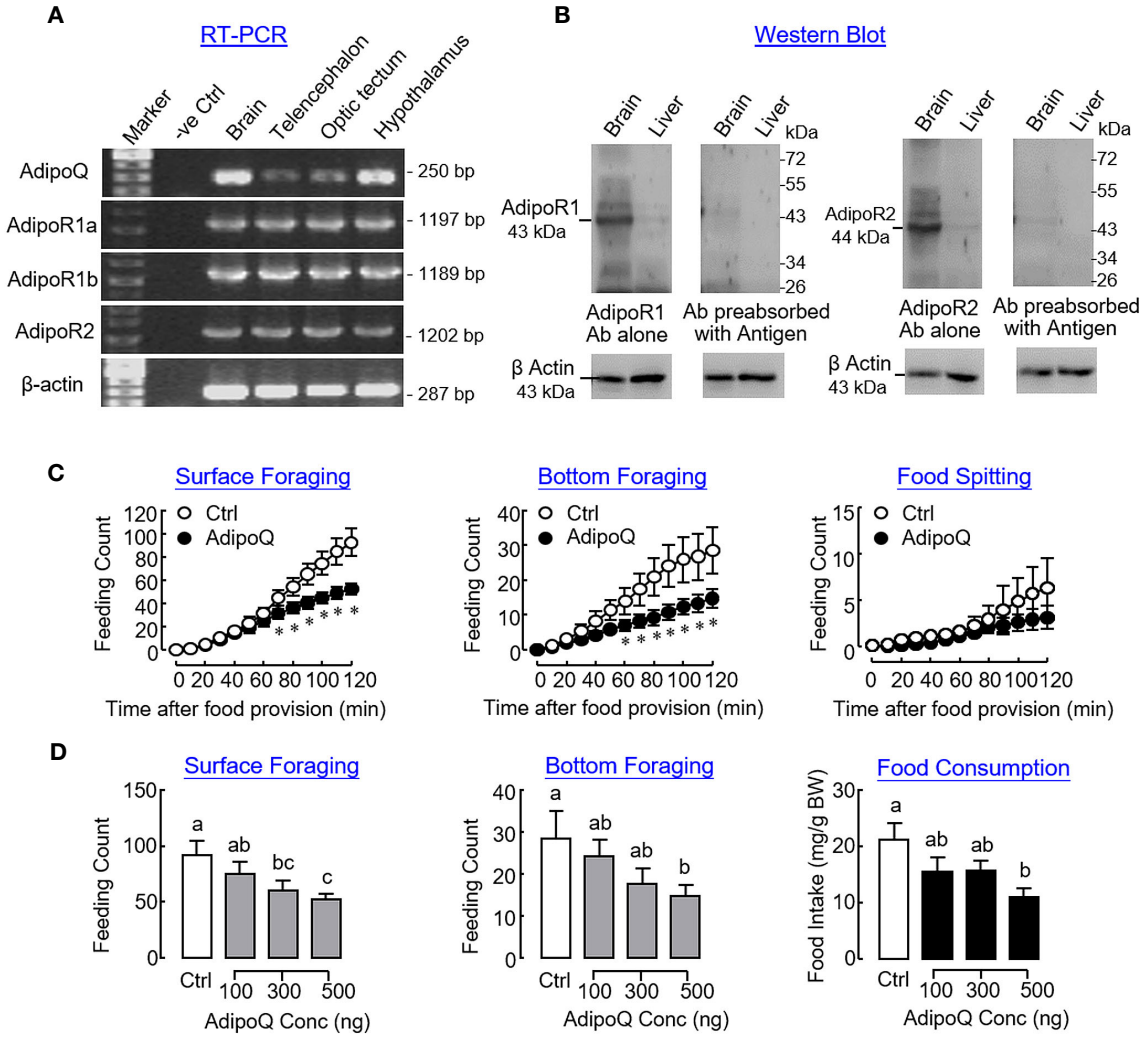
Expression of AdipoQ and its receptors within the brain and central actions of AdipoQ on feeding behaviors and food consumption in goldfish. **(A)** Co-localization of AdipoQ and its receptors AdipoR1a, AdipoR1b, and AdipoR2 in brain areas involved in feeding control as revealed by RT-PCR. In this study, the brain was used as the positive control and parallel PCR without template was used as a negative control. RT-PCR targeting β actin expression was used as the internal control. **(B)** Detection of AdipoR1 and R2 immunoreactivities in goldfish brain and liver using Western blot. Using the antibodies (Ab) raised against mouse AdipoR1 and R2, respectively, Western blot was conducted in tissue lysates prepared from the brain and liver of the goldfish, respectively. The specificity of AdipoR1/R2 signals detected was also confirmed by parallel Western blot using the same antibodies preabsorbed with the respective antigen peptides used to raise the two antibodies provided by the company. In this study, Western blot for β actin was conducted to serve as a loading control. **(C)** Time course and **(D)** dose dependence of central administration with AdipoQ on surface foraging, bottom foraging, and food spitting in goldfish. ICV injection of recombinant AdipoQ (300 ng/g BW for time course and 100–500 ng/g BW for dose dependence) was conducted 15 min prior to the scheduled feeding time, and different types of feeding behaviors were scored over a 2-h period after the introduction of food pellets. Parallel ICV injection of physiological saline was used as the control treatment. In the study for dose dependence of AdipoQ treatment, food consumption was also monitored by the end of the 2-h period. Groups denoted by asterisks or with different letters represent a significant difference at *p* < 0.05.

In goldfish, transcript signals of AdipoQ and its receptors AdipoR1a, AdipoR1b, and AdipoR2 could be detected in the brain and located in the telencephalon, optic tectum, and hypothalamus as revealed by RT-PCR (**Figure 8A**). Using Western blot, immunoreactivities for AdipoR1 and R2 were also detected in the brain and liver respectively, with the brain as the site with a high level of AdipoQ receptor expression (**Figure 8B**). To confirm the central action of AdipoQ in feeding control, ICV injection of AdipoQ (500 ng/fish) was conducted to test its effects on different types of feeding behaviors in goldfish. Similar to the results of our preceding study based on IP injection, central administration of AdipoQ was shown to reduce surface foraging and bottom foraging but not food spitting in a time-dependent manner (**Figure 8C**). In a parallel study with drug treatment fixed at 2 h, ICV injection with increasing levels of AdipoQ (100–500 ng/fish) was effective in triggering a dose-dependent inhibition on the two types of foraging activity with a concurrent drop in food consumption during the same period (**Figure 8D**).

### 3.8 ICV injection of AdipoQ on central expression of feeding regulators and their receptors

**Figure 9:**
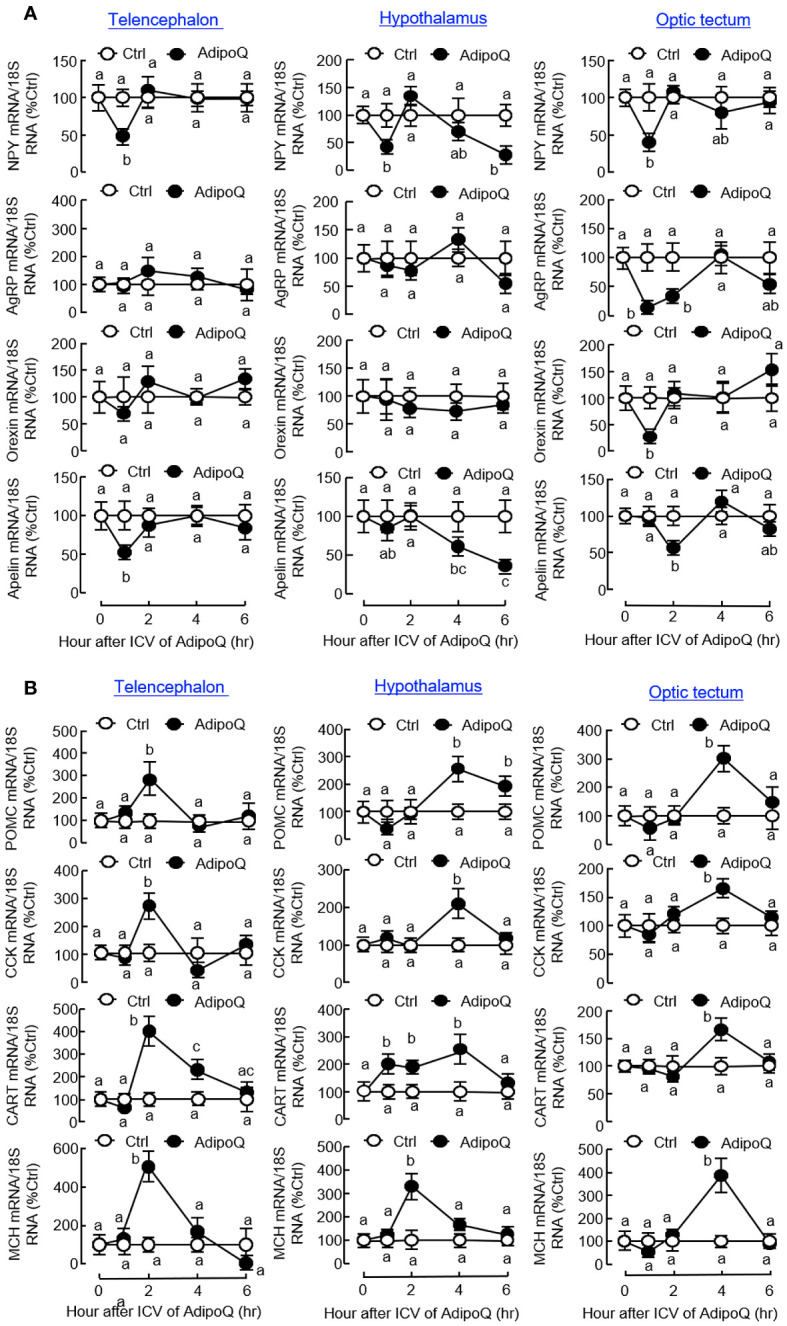
ICV injection of AdipoQ on central expression of orexigenic/anorexigenic factors in brain areas for feeding control in goldfish. Goldfish with prior training of “one-meal-per-day” feeding schedule were ICV injected with recombinant AdipoQ (300 ng/g BW) 15 min prior to the scheduled feeding time without food provision (as time zero). Parallel treatment with physiological saline was used as the control. After ICV injection, the brain areas including the telencephalon, hypothalamus, and optic tectum were harvested at the time points as indicated. Total RNA was isolated and subjected to real-time PCR for transcript expression of **(A)** orexigenic factors including NPY, AgRP, Orexin, and Apelin, and **(B)** anorexigenic factors including POMC, CCK, CART, and MCH. Real-time PCR for 18S RNA was also conducted to serve as the internal control. Groups denoted by different letters represent a significant difference at *p <* 0.05.

To decipher the mechanisms mediating the central actions of AdipoQ in feeding control, ICV injection of AdipoQ was performed in goldfish to examine its effects on central expression of feeding regulators and their receptors in different brain areas. As shown in **Figure 9A**, transient drops of NPY transcript were noted in the telencephalon, hypothalamus, and optic tectum with peak at 1 h after AdipoQ injection. Interestingly, after a brief recovery during 2–4 h, a secondary drop in NPY gene expression was observed by the end of 6 h in the hypothalamus but not in other brain areas examined. In addition, the inhibition on NPY expression and downregulation of the apelin transcript also occurred in the telencephalon and optic tectum (with transient inhibition during the first 1–2 h) as well as in the hypothalamus (with a delayed inhibition during 4–6 h). Meanwhile, transient drops in AgRP and orexin transcripts with peak at 1 h could also be observed in the optic tectum but not in the telencephalon and hypothalamus. Unlike the inhibitory effects for orexigenic factors, the anorexigenic factors examined were highly responsive to AdipoQ with notable stimulation in the telencephalon, hypothalamus, and optic tectum (**Figure 9B**). In these brain areas, ICV injection of AdipoQ was effective in inducing transient rises in POMC, CCK, CART, and MCH transcripts with peak stimulation occurred during 2–4 h. In the hypothalamus, the CART response was prolonged with a rapid onset at 1 h and lasted up to 4 h.

**Figure 10:**
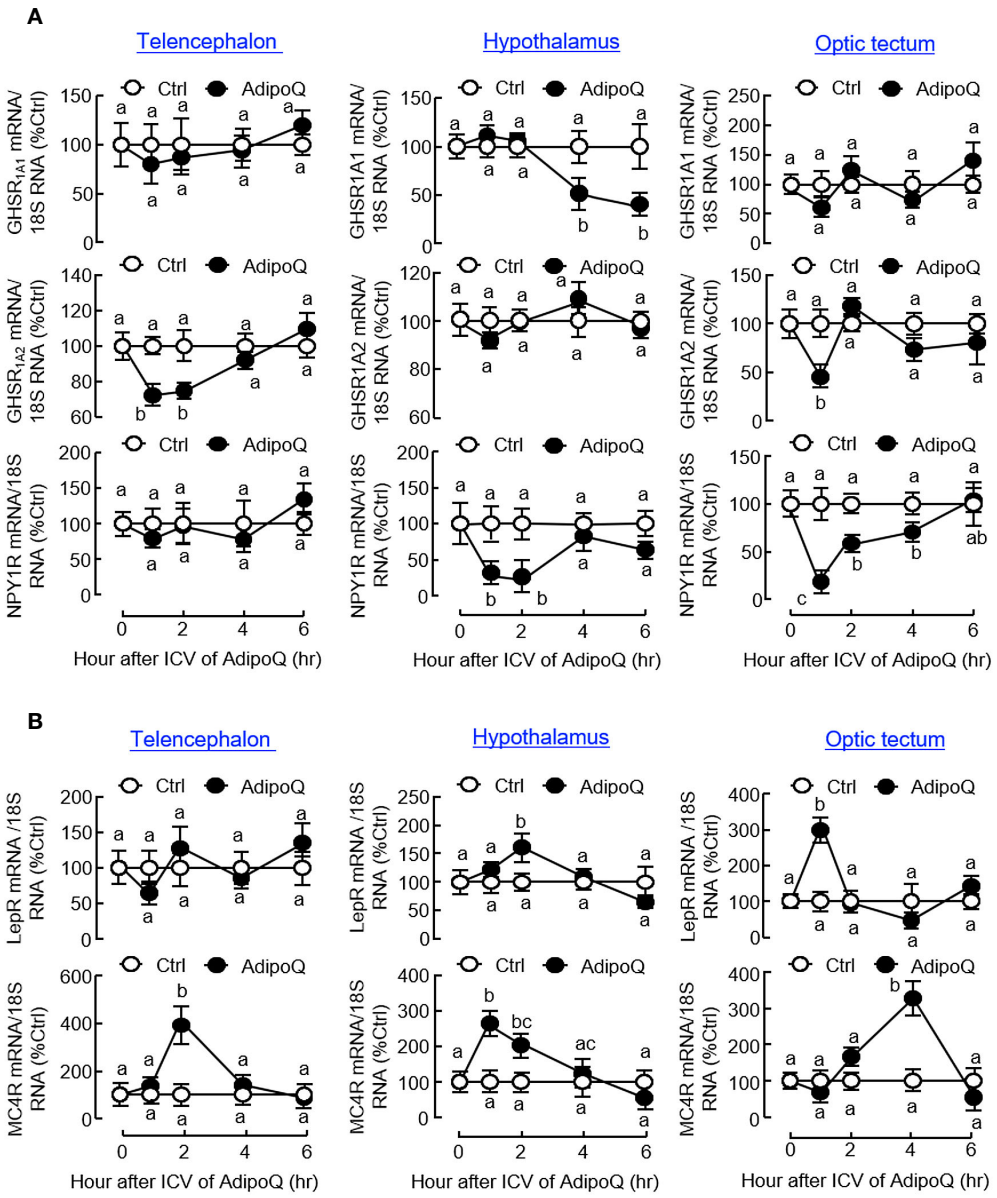
ICV injection of AdipoQ on receptor expression for orexigenic/anorexigenic signals in brain areas for feeding control in goldfish. ICV injection of AdipoQ (300 ng/g BW) was performed as described in preceding figure with parallel treatment of physiological saline as the control. After that, brain areas including the telencephalon, hypothalamus, and optic tectum were harvested at the time points as indicated. Total RNA was isolated and subjected to real-time PCR for transcript expression of **(A)** the receptors for orexigenic signals including GHSR_1A1_, GHSR_1A2_, and NPY1R, and **(B)** the receptors for anorexigenic signals including LepR and MC4R. Real-time PCR for 18S RNA was used as internal control, and groups denoted by different letters represent a significant difference at *p <* 0.05.

Similar to the results based on IP injection, central administration of AdipoQ also induced differential expression of the receptors for various feeding regulators. Regarding the receptors for orexigenic factors (**Figure 10A**), transient drops in transcript expression for GHSR_1A2_ in the telencephalon and optic tectum and NPY1R in the hypothalamus and optic tectum (both with peak inhibition during 1–2 h) could be observed after ICV injection of AdipoQ. Downregulation of GHSR_1A1_ was also detected in the hypothalamus. In this case, unlike the rapid responses for GHSR_1A2_ in other brain areas, the inhibition on GHSR_1A1_ expression was much delayed in the hypothalamus with gradual reduction of the GHSR_1A1_ transcript occurring during 4–6 h after AdipoQ treatment. Regarding the receptors for anorexigenic factors (**Figure 10B**), similar to the results based on IP injection, transcript levels of LepR were transiently elevated in the hypothalamus (with peak at 2 h) and optic tectum (with peak at 1 h) after ICV injection of AdipoQ. Although IP injection of AdipoQ did not alter MC4R expression in the brain areas examined, central administration of AdipoQ was effective in inducing notable stimulation on transcript expression of MC4R in the telencephalon (with peak at 2 h), hypothalamus (with peak at 1 h) and optic tectum (with peak at 4 h), respectively.

## 4 Discussion

AdipoQ is an adipokine with antidiabetic, anti-obesogenic, and anti-atherogenic actions (27) and known to have a protective role for obesity, diabetes, atherosclerosis, and cardiovascular disease (2, 23). Since the beneficial effects of AdipoQ on these pathological conditions can be partly attributed to its regulation on body weight via appetite modulation, feeding control by AdipoQ and the mechanisms involved have been a major topic for AdipoQ research in recent decades. However, the results obtained based on mammalian models (e.g., in rodents) are highly controversial if not contradictory and the cause for the discrepancies is still unclear (35). To shed light on the comparative aspects of AdipoQ as a feeding regulator, goldfish was used as a model to examine the functional association of AdipoQ with food intake and feeding regulation in lower vertebrates. As a first step, the structural identity of goldfish AdipoQ was established by molecular cloning. Based on phylogenetic analysis of the sequence obtained, goldfish AdipoQ was confirmed to be a member of the fish AdipoQ family and closely related to zebrafish AdipoQ. Data mining of the full gene for goldfish AdipoQ from the NCBI database with parallel comparison with AdipoQ genes in other vertebrate species revealed that the intron/extron organization of the AdipoQ gene (with two introns and three exons) has been well conserved from fish to mammals (except for human AdipoQ gene with an additional intron inserted into the original exon 1). As shown by the results of comparative synteny using the neighboring genes found upstream/downstream of AdipoQ genes in different species, the gene for AdipoQ probably had gone through two rounds of genomic translocation during vertebrate evolution, with the first round occurred with the AdipoQ gene alone during the evolution from fish to amphibians and the second round occurred in the gene fragment carrying AdipoQ and ST6GAL1 genes during the evolution of the mammalian lineage. Our findings, to our knowledge, represent the first to report the genomic relocation of the AdipoQ gene during the key steps of vertebrate evolution.

Similar to the protein structure of AdipoQ in other species, the precursor protein encoded by the ORF of goldfish AdipoQ is composed of a signal peptide in the N-terminal linked with a species-specific variable region followed by the highly conserved collagen-like domain and globular domain in the C-terminal. Using multiple-sequence alignment of goldfish AdipoQ with the AdipoQ in other species, the Cys^55^ residue located close to the end of the variable region (equivalent to Cys^36^ in human and Cys^39^ in mouse AdipoQ), the 22 G-X-Y repeats with the Lys^87^, Lys^96^, and Lys^120^ residues for hydroxylation/glycosylation in the collagen-like domain (equivalent to Lys^68^, Lys^77^, and Lys^101^ in human AdipoQ), and the 10 anti-parallel β-sheet motifs spreading within the globular domain were confirmed to be well-conserved from fish to mammals. Parallel 3D protein modeling also revealed that the spatial clustering of the 10 anti-parallel β-sheets to form the globular domain and regular spacing of the 20 G-X-Y repeats in the collagen-like domain were highly comparable if not identical between the goldfish and human AdipoQ. Based on the studies in mammalian AdipoQ, the conserved residue of Cys located close to the end of the variable region is involved in disulphide bond formation, which is essential for AdipoQ multimerization to form the MMW and HMW isoforms (53) and plays a role in AdipoQ release via modulation of thiol-mediated protein retention (54). The conserved Lys residues in the collagen-like domain also contribute to AdipoQ multimerization via hydroxylation and glycosylation with subsequent modification of the bioactivity of the final AdipoQ products (e.g., enhancing the insulin-sensitizing effect of AdipoQ in rat hepatocytes) (55). The G-X-Y repeats of the collagen-like domain are known to be crucial in the formation of the triple-helical structure commonly found in collagen fibres/C1q proteins, and the same triple helix can stabilize the trimeric form of AdipoQ to yield the typical “ball-on-a-stick” structure of AdipoQ trimer (56). In a recent study, a 13-a.a. fragment within the collagen-like domain composed of multiple GXXG motifs formed by G-X-Y repeats was shown to enhance AdipoQ oligomerization and induce AMPK activation in rat L6 myoblasts (57). In addition to the triple helix formed by “twisting” of the collagen-like domains, the anti-parallel β-sheets within the globular domain by adopting a 10-strand jelly-roll β barrel topography with a hydrophobic surface at the base (to allow for non-covalent intramolecular interactions), and Ca^2+^ trapping in the apex region (to rigidify the apical loops of AdipoQ trimer) can also allow for a tight packaging of the “globular head” in the AdipoQ trimer (52). As a whole, the structural conservation of the signature motifs and functional residues found in goldfish AdipoQ compared with its counterparts in higher vertebrates implies that AdipoQ has evolved under a strong selective pressure, which is consistent with its involvement in a wide spectrum of important biological functions reported in the literature (for details, see introduction).

In mammals, AdipoQ is produced mainly by visceral fat (especially in mature adipocytes) (58) and with low level of expression in other tissues, including the eye (59), placenta (60), and various tissues within the reproductive system (61). Adipose tissue as the major site for AdipoQ expression has also been reported in avian species, e.g., chicken (62). In our study, AdipoQ was found to be ubiquitously expressed in goldfish with high levels in the heart and muscle, to a lower extent in the gill, testes, and brain, and with low levels in the liver, kidney, spleen, ovary, and fat. Our results on tissue expression of AdipoQ are quite comparable with the reports in rainbow trout (40), yellow croaker (42), and sturgeon (43), in which the heart and/or muscle represent the major tissues with the highest level of AdipoQ expression. Since AdipoQ is expressed at a low level/barely detectable in the adipose tissue of zebrafish similar to that of goldfish (39), we do not exclude the possibility that AdipoQ may act as a myokine rather than adipokine in fish models. Within the brain in goldfish, the highest level of AdipoQ expression was located in the hypothalamus and with notable AdipoQ signals detected in the telencephalon and optic tectum. Given that these brain areas are known to contain the feeding circuitry for appetite control in fish species (63), we speculate that AdipoQ may play a role in feeding control in goldfish similar to that in mammals. To prepare for the functional studies to test our hypothesis, recombinant goldfish AdipoQ (expressed in the form of gAdipoQ) as well as its antiserum were produced. Based on our structural characterization using CD, SEC-MALS, and native PAGE under non-denaturing condition, the β-sheet structures in recombinant goldfish AdipoQ were properly folded within the globular domain and leading to the formation of trimeric AdipQ (also with low levels of hexameric and HMW forms). Similar to the studies of gAdipoQ in mammals, e.g., gAdipoQ-induced AMPK activation in rat muscle (64) and NF-κB activation in U937 monocytes (65), our AdipoQ preparation was confirmed to be bioactive in terms of activating the goldfish AdipoQ receptors expressed in HepG2 cells, which could lead to AMPK activation with subsequent inhibition of the transcriptional activity mediated by PPARγ in our Luc reporter system. For the antiserum raised against the recombinant AdipoQ prepared, it was shown to be a high-titre antiserum specific for goldfish AdipoQ and with no cross-reactivity with other hormones commonly found in fish blood. Our studies also revealed that the antiserum could be used for (i) AdipoQ detection in goldfish tissues using Western blot (e.g., in the liver) and (ii) quantitation of AdipoQ secretion in plasma samples from goldfish and grass carp using an FIA format. Apparently, the antiserum produced not only could be useful for our goldfish study but also have potential applications for AdipoQ research in related carp species (e.g., grass carp).

The functional role of AdipoQ in appetite control has been a major focus for biomedical research as the genetic variants in AdipoQ can be linked with modifications/abnormalities in human eating behavior (66) and notable changes in serum AdipoQ have been reported in patients with eating disorders, e.g., with elevated levels in patients with anorexia nervosa whereas the opposite is true for binge eating (67). For the feeding control by AdipoQ, the results published based on animal models (e.g., rodents) are controversial if not conflicting. For examples, ICV injection of AdipoQ in the rat (33) and mouse (38) has been reported to suppress food intake. In the rat, this inhibitory action was mediated by AdipoR1 within the hypothalamus coupled to the IRS_1_/Akt/FOXO_1_ and JAK_2_/STAT_3_ pathways (33). However, similar treatment with AdipoQ by ICV injection in the mouse model through AdipoR1 activation with subsequent AMPK signaling in the hypothalamus was found to increase food consumption by another research group (32). Using rodents as animal models, other studies have demonstrated that AdipoQ did not affect food intake, despite that AdipoQ treatment in the same study was effective in reducing body weight (e.g., in mice) (31) or improving glucose homeostasis with a parallel drop in fat storage (e.g., in rats) (34). In transgenic mice with GFP labeling of NPY and POMC neurons in the hypothalamus, AdipoQ was shown to have differential effects on food intake depending on glucose level in the CNS, with stimulation via AMPK-mediated inhibition of POMC neurons under high glucose but inhibition via PI3K-mediated activation of POMC neurons with low glucose (38). Interestingly, AdipoQ consistently reduced NPY signal via hyperpolarization of NPY neurons within the hypothalamus independent of glucose (37), which may contribute to the feeding inhibition observed in the mouse model. For the reports supporting feeding stimulation by AdipoQ (e.g., in mice), gene knockout of AdipoQ was shown to increase POMC with current drop in NPY gene expression in the hypothalamus (32). In another study with the same animal model, however, ICV injection of AdipoQ did not alter the central expression of NPY/POMC (31). At present, a common consensus has not been reached regarding the role as well as the underlying mechanisms for feeding control by AdipoQ. In fish models, AdipoQ has been cloned in a limit no of fish species, including four species of bony fish (Ayu, zebrafish, rainbow trout, and yellow croaker) and one species of cartilaginous fish (Siberian sturgeon) (39–43). However, only three reports in fish have touched on the association of AdipoQ with food intake/food deprivation. In zebrafish, fasting was shown to inhibit AdipoQ expression in the liver (39), but the opposite was true in adipose tissue of rainbow trout with food deprivation (40). In Siberian sturgeon, IP injection of AdipoQ could induce a marginal drop in food consumption with parallel inhibition of NPY and POMC expression in the hypothalamus (43). Since (i) conflicting results of fasting on tissue expression of AdipoQ were noted in different fish species and (ii) parallel drop in orexigenic and anorexigenic signals within the hypothalamus could be associated with the minor inhibition on food intake caused by AdipoQ in sturgeon, the functional role and mechanisms involved in feeding control by AdipoQ are still unclear in fish models. In our study with goldfish, food consumption could induce AdipoQ release into circulation and upregulate AdipoQ expression in the liver as well as in brain areas including the telencephalon, hypothalamus, and optic tectum. Meanwhile, IP and ICV injection of our recombinant AdipoQ preparation were both effective in reducing foraging behaviors and food consumption in a dose-dependent manner. These results imply that (i) AdipoQ signals (both central and peripheral) can be induced by feeding and (ii) AdipoQ by acting centrally (probably may also have peripheral effects) can inhibit food intake by suppressing feeding behaviors. Our findings, as a whole, suggest that AdipoQ may act as a satiety factor in goldfish and play a role in the satiation response observed after a meal. Using the newly validated FIA for goldfish AdipoQ, we have demonstrated for the first time that a postprandial rise in circulating signal of AdipoQ could be induced by feeding in parallel with the central expression of AdipoQ. Since a postprandial rise in AdipoQ expression in peripheral tissues was found only in the liver but not in other tissues examined, it would be logical to assume that the rise in circulating AdipoQ was originated from the liver. In rodents, AdipoQ receptors are expressed in the choroid plexus (68), a key area in the brain for the entry of circulatory proteins/hormones through the blood–brain barrier (e.g., by receptor-mediated transcytosis), and IP injection of AdipoQ in AdipoQ knockout mice can also elevate AdipoQ level in cerebrospinal fluid (32). Therefore, we do not exclude the possibility that the postprandial rise of AdipoQ in circulation may enter the blood–brain barrier and act with the AdipoQ signals produced locally within the brain to suppress the foraging activity and food intake in goldfish.

Goldfish has been widely used as a fish model to study feeding regulation in lower vertebrates, mainly due to their low maintenance cost compared with rodents and their well-conserved neuroendocrine system for appetite control (44). Although the brain nuclei/regulatory centers for feeding control are not fully identical between mammals and bony fish due to different patterns of embryonic development in the CNS (e.g., the evagination development in mammals versus the eversion development in fish during the formation of the forebrain), the key components for central regulation of feeding (e.g., the feedback circuitry with NPY and POMC neurons in the hypothalamus) are highly comparable (69). In goldfish, using electrical stimulation or selective lesioning in different brain areas, the inferior lobe of the hypothalamus together with the neural input from the telencephalon (with signals from olfactory bulbs) and the optic tectum (with signals from optic nerves) have been reported to be the brain areas for organization and regulation of feeding behaviors (70). Based on extensive studies, most of the neuroendocrine factors for appetite control reported in mammals have been identified and functionally confirmed in goldfish. For examples, the orexigenic factors NPY, AgRP, orexin, ghrelin, and apelin are known to increase food intake after IP/ICV injection in goldfish whereas the opposite is true for similar treatment with the anorexigenic factors αMSH, CCK, CART, and leptin [for review, see (71)]. Of note, MCH is well-documented to be an orexigenic factor in mammals (72) but it was found to inhibit food intake in goldfish after ICV injection (73). In goldfish, immunoneuralization by ICV injection with MCH antiserum could also elevate basal feeding (74), indicating that MCH may serve as an anorexigenic factor in fish models. In our study, AdipoQ treatment reduced foraging activity and food intake in goldfish. Parallel to the feeding inhibition, differential regulation of feeding regulators and their receptors in brain areas involved in feeding control was also observed. In this case, IP and ICV injection of AdipoQ were both effective in stimulating POMC, CCK, CART, and MCH with parallel drops in NPY, AgRP, orexin, and apelin expression in the telencephalon, hypothalamus, and/or optic tectum. Meanwhile, downregulation of the receptors for NPY (NPY1R) and ghrelin (GHSR_1A1 & 1A2_) with notable rise of the receptor for leptin (LepR) also occurred in these brain areas. Since our study in goldfish has demonstrated that (i) AdipoR1 and R2 immunoreactivities were detected in the brain; (ii) AdipoQ was co-expressed with its receptors (AdipoR1a, R1b & R2) in the telencephalon, hypothalamus, and optic tectum; and (iii) Adipo R1a, R1b, and R2 expressed in HepG2 cells could be activated by recombinant goldfish AdipoQ, it is likely that AdipoQ produced in the telencephalon, hypothalamus, and optic tectum via AdipoR1/R2 activation can induce differential effects on central expression of orexigenic/anorexigenic signals with parallel changes in the sensitivity to different feeding regulators via modulation of their receptors expressed in the respective brain areas.

In our study, the results obtained based on IP injection versus ICV injection of AdipoQ for differential regulation of orexigenic/anorexigenic factors and selected receptors were highly comparable (especially in the hypothalamus and optic tectum) but still with variations in terms of the time course and brain areas with the respective responses. Of note, in contrast to a lack of response for the melanocortin receptor MC4R with IP injection, ICV injection with AdipoQ was effective in triggering a transient but notable rise in MC4R expression in all the three brain areas examined, implying that AdipoQ by acting directly on the feeding centers within the CNS can also enhance the sensitivity to feeding inhibition caused by the POMC gene product αMSH. The discrepancies observed between the results based on IP versus ICV injection of AdipoQ probably were caused by the peripheral actions of AdipoQ. This idea is supported by our findings that IP injection of AdipoQ could upregulate leptin A1 and A2 with a concurrent drop in ghrelin expression in the liver. In mammals, leptin and ghrelin are known to have an opposite effect on food intake, with ghrelin as the “hunger hormone” to initiate feeding and leptin as a satiety factor to reduce food intake after a meal, and genetic variants/mutations in these two peripheral factors can be associated with observable changes in feeding behavior (75). Based on the studies in rodents, ghrelin via GHSR activation can activate the hypothalamic neurons with NPY, AgRP, and orexin expression with parallel inhibition on the neurons with POMC and CRH expression (76, 77), and the opposite is true for leptin modulation of NPY, AgRP, orexin, POMC, and CRH neurons in the same area (76, 78). In the hypothalamus (e.g., rats), leptin can also inhibit orexin receptor expression caused by fasting (79), implying that the peripheral feeding regulators can exert an intricate interaction with the central feeding signals in the brain. In goldfish, food intake induced by ICV injection of ghrelin could be blocked by co-treatment with the NPY1R antagonist (80) or orexin antagonist (81), indicating that the orexigenic effect of ghrelin is mediated by central expression of NPY and orexin. Furthermore, IP injection of leptins A1 and A2 in goldfish not only could reduce food intake but also trigger differential regulation of NPY, AgRP, POMC, CART, and MCH in brain areas including the telencephalon, hypothalamus, and optic tectum (82). These findings, as a whole, support the notion that the changes in hepatic signals of leptin and ghrelin caused by AdipoQ may exert a secondary effect acting within the CNS to modify the expression of feeding regulators and their receptors in the brain areas examined.

**Figure 11:**
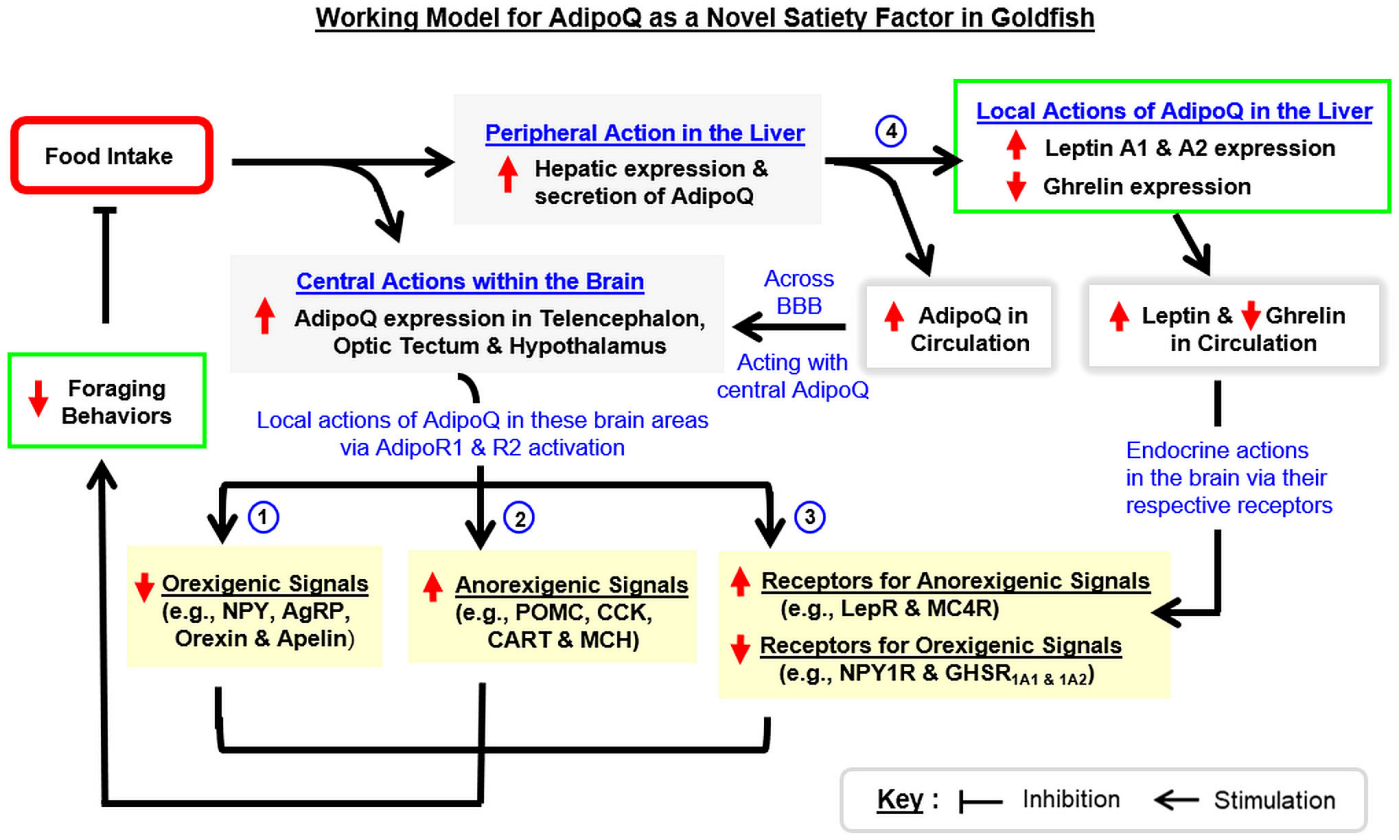
Working model for AdipoQ as a novel satiety factor in goldfish. In goldfish, food intake triggers AdipoQ signals both in the liver and within the brain. For the central responses, food intake can stimulate AdipoQ expression in brain areas involved in feeding control including the telencephalon, optic tectum, and hypothalamus. Through AdipoR1/R2 activation, AdipoQ expressed in these brain areas acts locally to (i) upregulate orexigenic signals including NPY, AgRP, orexin, and apelin, (ii) suppress anorexigenic signals including POMC, CCK, CART, and MCH, and (iii) differentially regulate receptor expression of orexigenic/anorexigenic signals, with a rise in the receptors mediating feeding inhibition (LepR and MC4R) but a drop in the receptors for feeding stimulation (NPY1R and GHSR_1A1 & 1A2_). In addition to the central actions, food intake can also induce AdipoQ expression in the liver with a subsequent rise of AdipoQ secretion into systemic circulation. The endocrine signal of AdipoQ probably can pass through the blood–brain barrier (BBB) and act together with the central signals of AdipoQ to suppress different types of foraging behaviors observed in goldfish. Meanwhile, AdipoQ produced at the hepatic level may act locally to stimulate leptin A1 and A2 expression with a concurrent drop in ghrelin expression in the liver. The corresponding changes in leptin and ghrelin output from the liver via subsequent endocrine actions in the brain may further enhance/prolong the feeding inhibition caused by AdipoQ signals.

To summarize, we have cloned and characterized the structural and evolutionary aspects of goldfish AdipoQ with parallel mapping of its tissue distribution. Based on the sequence obtained, recombinant AdipoQ with bioactivity to activate goldfish AdipoR1a, R1b, and R2 was produced and used to raise the antiserum for goldfish AdipoQ with subsequent establishment of an FIA for measuring AdipoQ release in goldfish plasma. These new tools were then used to examine the association of AdipoQ with food intake and feeding regulation in goldfish. In our study, food intake in goldfish could induce AdipoQ release in circulation with parallel rises of AdipoQ expression in the liver and brain areas including the telencephalon, hypothalamus and optic tectum. Interestingly, IP/ICV injection with recombinant AdipoQ was effective in inhibiting foraging activity and food intake in goldfish. Meanwhile, AdipoQ was found to differentially regulate the feeding regulators (with up-regulation of POMC, CART, CCK, and MCH and down-regulation of NPY, AgRP, orexin, and apelin) and related receptors (with upregulation of MC4R and LepR and downregulation of NPY1R and GHSR_1A1 & 1A2_) in the telencephalon, hypothalamus, and/or optic tectum. In addition to the central action, IP injection of AdipoQ also increased leptin A1 and A2 with a parallel drop in ghrelin expression in the liver. Based on the results obtained, a working model (**Figure 11**) has been proposed for the mechanisms underlying the feeding control by AdipoQ. In this model, food intake in goldfish can induce AdipoQ signals of the peripheral (from the liver) and central origin (in the telencephalon, hypothalamus, and optic tectum). These postprandial signals of AdipoQ via AdipoR1/R2 activation can act directly within the respective brain areas to exert a negative feedback to suppress foraging behaviors and subsequent food consumption. Apparently, the process involves AdipoQ modulation of feeding regulators (both orexigenic and anorexigenic) and related receptors (with orexigenic/anorexigenic actions) expressed in the same brain areas. Parallel to these central effects, AdipoQ produced in the liver via autocrine/paracrine actions can also induce differential expression of leptin and ghrelin at the hepatic level, and the subsequent rise in leptin output with concurrent drop in ghrelin signal in systemic circulation presumably can prolong or even enhance the satiation response after a meal in goldfish. Our findings, as a whole, provide evidence for the first time that AdipoQ can act as a novel satiety factor in a fish model. Given that the comparative aspects of AdipoQ in the satiation response after a meal are still lacking, it will be of interest to extend our study to mammalian models (e.g., in rodents) to see if AdipoQ can also serve as a satiety factor in higher vertebrates, which for sure will have biomedical implications in appetite control and human obesity.

## Data availability statement

All the data for our study are presented in the figures and supplementary materials of this paper. The sequences of grass carp AdipoQ (Accession no ON087697), AdipoR1a (Accession no OQ447502), AdipoR1b (Accession no OQ447503) and AdipoR2 (Accession no OQ447504) have been submitted to Genbank and can be download from https://www.ncbi.nlm.nih.gov/genbank/.

## Ethics statement

The animal study was approved by the Committee on the Use of Live Animal for Teaching & Research, the University of Hong Kong (Hong Kong). The study was conducted in accordance with the local legislation and institutional requirements.

## Author contributions

YZ: Investigation, Data curation, Visualization. CY: Formal analysis, Software. MH: Investigation, Data curation, Visualization. WK: Methodology, Data curation, Visualization. YC: Resources, Supervision. AW: Conceptualization, Funding acquisition, Project administration, Supervision, Writing – original draft, Writing – review & editing.

## Glossary

**Table:**

AdipoQ	Adiponectin
AdipoR1 & R2	Adiponectin type 1 & 2 receptors
NPY	Neuropeptide Y
AgRP	Agouti-related peptide
CCK	Cholecystokinin
CART	Cocaine- and amphetamine-regulated transcript
MCH	Melanin-concentrating hormone
POMC	Pro-opiomelanocortin
GH	Growth hormone
PRL	Prolactin
SL_α/β_	Somatolactin α/β
NPY1R	Type 1 NPY receptor
GHSR_1A1/1A2_	Ghrelin receptor subtype 1A1/1A2
LepR	Leptin receptor
MC4R	Melancortin type 4 receptor
CD	Circular dichroism
PAQR	Progestin/AdipoQ receptor
IMAC	Immobilized metal affinity chromatography
SEC	Size exclusion chromatography
SEC-MALS	SEC with multi-angle laser light scattering analysis
AMPK	AMP-activated protein kinase
IP injection	Intraperitoneal injection
ICV injection	Intracerebroventricular injection
FIA	Fluorescence immunoassay
RT-PCR	Reverse transcription PCR
RACE	Rapid amplifiation of cDNA ends
ORF	Open reading frame
5’/3’ UTR	5’/3’ Untranslated region
CNS	Central nervous system
