# Supplementary material for: Goldfish adiponectin: (I) molecular cloning, tissue distribution, recombinant protein expression, and novel function as a satiety factor in fish model
Source: Front Endocrinol (Lausanne). 2023 Oct 30;14:1283298. doi: 10.3389/fendo.2023.1283298 (PMC10643153; doi:10.3389/fendo.2023.1283298)
Supplement: Supplementary file 1 [file DataSheet_1.pdf]

Supplementary Table.1 Primers and PCR conditions for RT-PCR for selected gene targets

Gene Target / GenBank Accession No.<br>Sequences of Forward (F) & reverse primers (R)	PCR condition				Product size
	Denaturing	Annealing	Extension	Cycle	
AdipoQ / ON087697<br>F: 5'- GTGCCAATCCGCTTCAACA -3'<br>R: 5'- CCTCATCTCCTGCCTCCAAA -3'	94°C<br>30 sec	62°C<br>30 sec	72°C<br>30 sec	x 35	250 bp
AdipoR1a / OQ447502<br>F:5'- ATGTCAGGCCAAATCAGGTCTG-3'<br>R:5'- GATGACCATGTAGGAGGTAGTCATTG -3'	94°C<br>30 sec	59°C<br>30 sec	72°C<br>70 sec	x 35	1197 bp
AdipoR1b / OQ447503<br>F: 5'- ATGACAACGTGTCACCATGGTGAC -3'<br>R: 5'-CTGGGAGGAGATGGTATGGAATAACAC-3'	94°C<br>30 sec	60°C<br>30 sec	72°C<br>70 sec	x 35	1189 bp
AdipoR2 / OQ447504<br>F: 5'- ATGAGTGCCAGCACAGATCACAG -3'<br>R: 5'- CTGAAGATGCTCTTGAAACAAGCTCTG-3'	94°C<br>30 sec	60°C<br>30 sec	72°C<br>70 sec	x 35	1202 bp
β-actin / AB039726.2<br>F: 5'- CTGGTATTCGTGATGGACTCT -3'<br>R: 5'- AGCTCATAGCTCTTCTCCAG -3'	94°C<br>30 sec	56°C<br>30 sec	72°C<br>30 sec	x 35	287 bp
