# Supplementary material for: Goldfish adiponectin: (I) molecular cloning, tissue distribution, recombinant protein expression, and novel function as a satiety factor in fish model
Source: Front Endocrinol (Lausanne). 2023 Oct 30;14:1283298. doi: 10.3389/fendo.2023.1283298 (PMC10643153; doi:10.3389/fendo.2023.1283298)
Supplement: Supplementary file 2 [file DataSheet_2.pdf]

Supplementary Table.2 Primers and PCR conditions for real-time PCR for selected gene targets

Gene Target / GenBank accession No. Sequences of Forward (F) & Reverse primers (R)	Real-time PCR condition					Product size & T_m value
	Denaturing	Annealing	Extension	Detection	Cycle	
AdipoQ / ON087697<br>F: 5′- GTGCCAATCCGCTTCAACA-3′<br>R: 5′- CCTCATCTCCTGCCTCCAAA-3′	94°C 30 sec	62°C 30 sec	72°C 30 sec	83°C 20 sec	x 35	250 bp Tm = 88 °C
NPY / M87297<br>F: 5′-GTAGTGTTGCGGGTAGCGA-3′<br>R: 5′-CAGACACCCCGACCCAAG-3′	94°C 30 sec	64°C 30 sec	72°C 30 sec	88°C 20 sec	x 35	234 bp Tm = 92 °C
AgRP / AJ555492<br>F: 5′-TGGCATCACATCCAAACCT-3′<br>R: 5′-CAGGTGATGACCCAAGCAG-3′	94°C 30 sec	64°C 30 sec	72°C 30 sec	82°C 20 sec	x 35	230 bp Tm = 82 °C
Orexin / DQ923590<br>F: 5′-GCAGAGCTGC-CATTGTTGACGTT-3′<br>R: 5′-AACCTTGTGATTACCTCAGGAGT-3′	94°C 30 sec	64°C 30 sec	72°C 30 sec	79°C 20 sec	x 35	286 bp Tm = 82 °C
Apelin / FJ755698<br>F: 5′-GAGCATAGCAAAGAGCTGGA-3′<br>R: 5′-AACCTTGTGATTACCTCAGGAGT-3′	94°C 30 sec	64°C 30 sec	72°C 30 sec	89°C 20 sec	x 35	340 bp Tm = 94 °C
POMC / AJ431209<br>F: 5′-AAGCGCTCCTACTCCATGGA-3′<br>5′-CTCGTCCCAGGACTTCATGAA-3′	94°C 30 sec	60°C 30 sec	72°C 30 sec	83°C 20 sec	x 35	282 bp Tm=85 °C
CART / AF288810<br>F: 5′-CCAAAGGACCCGAATCTGA-3′<br>5′-TITGCCGATTCTTGACCCT-3′	94°C 30 sec	64°C 30 sec	72°C 30 sec	72°C 20 sec	x 35	171 bp Tm = 90 °C
CCK / CAU70865<br>F: 5′-CCGCAGTCTCAGAAGATGGG-3′<br>5′-GGAGGGGCTTCTGCGATA-3′	94°C 30 sec	64°C 30 sec	72°C 30 sec	87°C 20 sec	x 35	197 bp Tm = 91 °C
MCH / AM403730<br>F: 5′-AGGCTTGAGCGAGAACTTGG-3′<br>R: 5′-CCCAGAAGACCTACACCTCCC-3′	94°C 30 sec	64°C 30 sec	72°C 30 sec	86°C 20 sec	x 35	272 bp Tm = 91 °C
NPY1R / XM_026204017.1<br>F: 5′-CTCGGACAGCCATGTAGACC-3′<br>R: 5′-TCTGGCATTGTTCCACCTCC-3′	94°C 30 sec	67°C 30 sec	72°C 30 sec	89°C 20 sec	x 35	254 bp Tm = 88 °C
$GHSR_{1A1}$ / AB504275<br>F: 5′-TAAATGTTGAGCAGCCCTTCGCGC-3′<br>R: 5′- GGGCATGCAGAGAAAAATAAA-3′	94°C 30 sec	65°C 30 sec	72°C 30 sec	85°C 20 sec	x 35	380 bp Tm = 92 °C
$GHSR_{1A2}$ / AB504276<br>F: 5′-ACAGGTTGTATAAGTTGAGCG-3′<br>R: 5′-AGGCATGCAGAGAAAAATGAG-3′	94°C 30 sec	65°C 30 sec	72°C 30 sec	86°C 20 sec	x 35	373 bp Tm = 90 °C
MC4R / AJ534337<br>F: 5′-TGGACCGCATCATTCATAC-3′<br>R: 5′-CAACAGTGAGCTGCAGATC-3′	94°C 30 sec	65°C 30 sec	72°C 30 sec	83°C 20 sec	x 35	376 bp Tm = 87 °C
Leptin receptor / EU911005<br>F: 5-CTGGCTTGAAGGTGAACGGAC-3′<br>R: 5-TTGGGTGACAGTGCAGTAGTC-3′	94°C 30 sec	65°C 30 sec	72°C 30 sec	78°C 20 sec	x 35	156 bp Tm = 87 °C
Leptin A1 / FJ534535<br>F: 5-TCCAAAGCTCCTCATAGG-3′<br>R: 5-TGGTGGGTGGCGTTTTCC-3′	94°C 30 sec	50°C 30 sec	72°C 30 sec	86°C 20 sec	x 35	270 bp Tm = 89 °C
Leptin A2 / FJ854572<br>F: 5-TATCGTGGACACCCTAACTAC-3′<br>R: 5-GGTCTAAAGCCAAGAACCCTAA-3′	94°C 30 sec	50°C 30 sec	72°C 30 sec	85°C 20 sec	x 35	224 bp Tm = 89 °C
Ghrelin / AF454389<br>F: 5′-GTAGTGTTGCGGGTAGCGA-3′<br>R: 5′-CAGACACCCCGACCCAAG-3′	94°C 30 sec	64°C 30 sec	72°C 30 sec	75°C 20 sec	x 35	471 bp Tm = 81 °C
18S RNA / HQ615531<br>F: 5′-AGCAACTTTAGTATACGCTATTGGA G-3′<br>R: 5′-CCTGAGAAACGGCTACCACATCC-3′	94°C 30 sec	56°C 30 sec	72°C 30 sec	87°C 20 sec	x 35	285 bp Tm = 91 °C
