# Supplementary figures and images for: Goldfish adiponectin: (I) molecular cloning, tissue distribution, recombinant protein expression, and novel function as a satiety factor in fish model
Source: Front Endocrinol (Lausanne). 2023 Oct 30;14:1283298. doi: 10.3389/fendo.2023.1283298 (PMC10643153; doi:10.3389/fendo.2023.1283298)

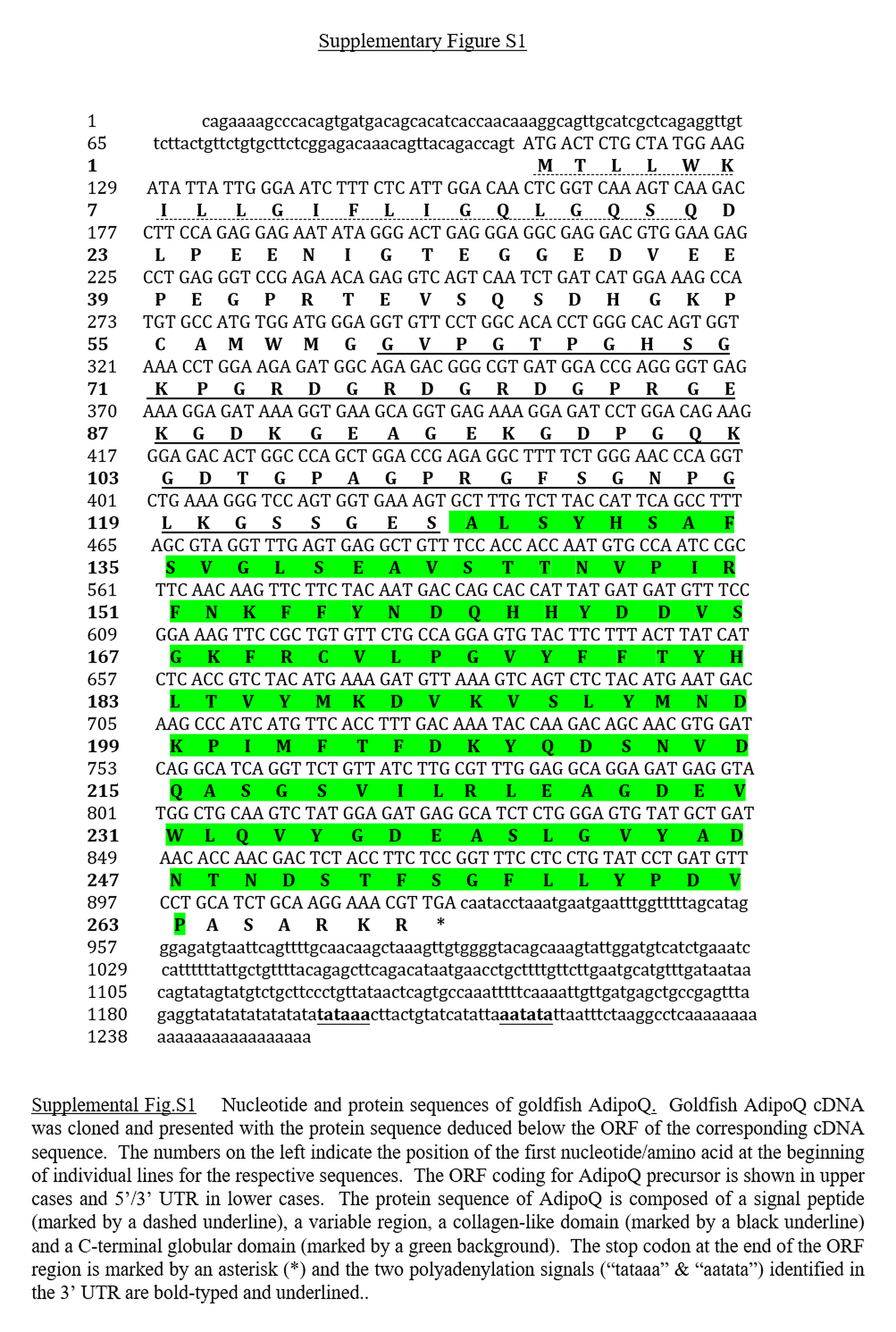

Supplement: Supplementary file 3 [file Image_1.tif]

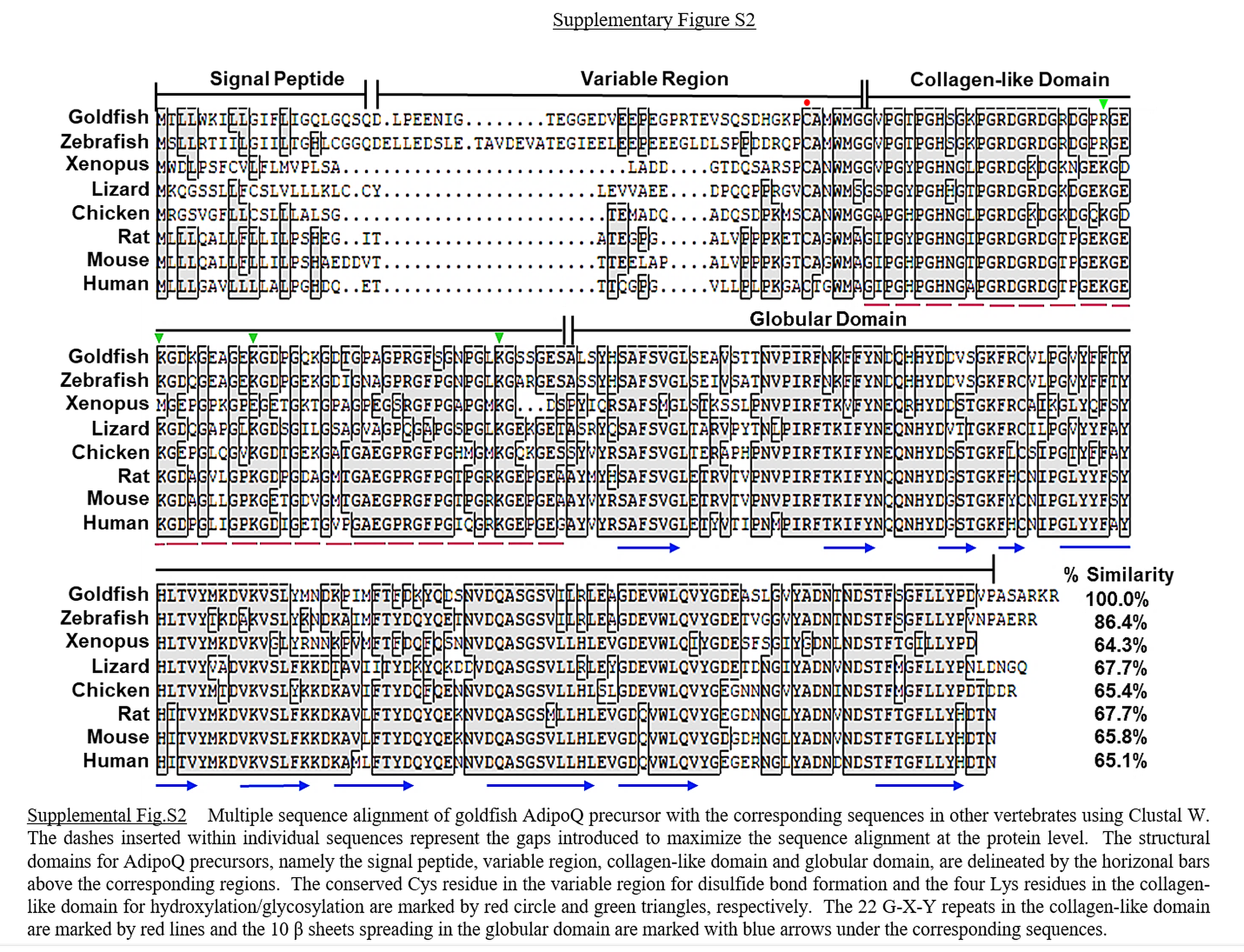

Supplement: Supplementary file 4 [file Image_2.tif]

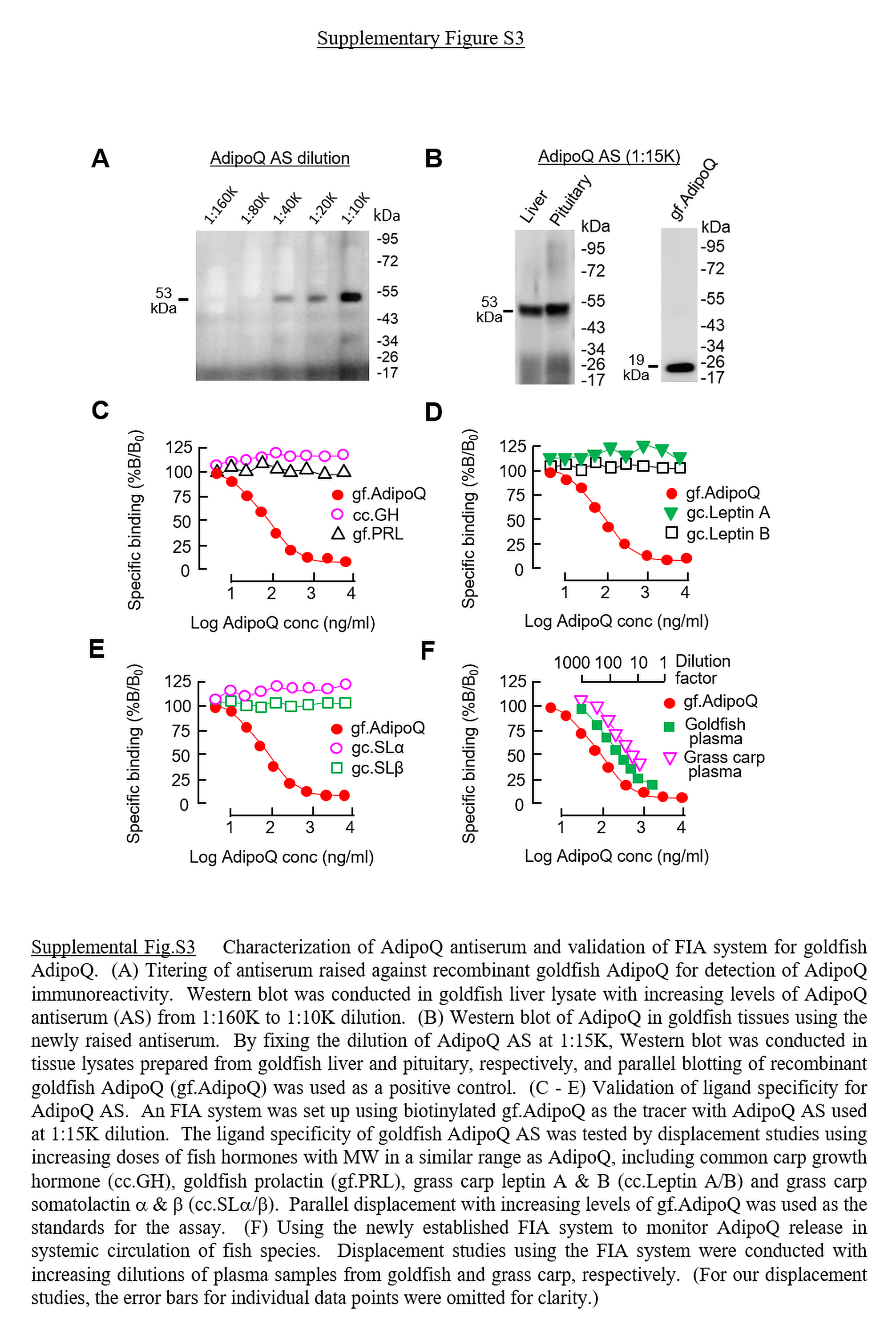

Supplement: Supplementary file 5 [file Image_3.tif]
